# Somatic Sex: On the Origin of Neoplasms With Chromosome Counts in Uneven Ploidy Ranges

**DOI:** 10.3389/fcell.2021.631946

**Published:** 2021-08-04

**Authors:** Oskar A. Haas

**Affiliations:** St. Anna Children’s Cancer Research Institute, Vienna, Austria

**Keywords:** aneuploidy, neoplasms, nanotubes, cell fusion, embryonic, genetic predisposition

## Abstract

Stable aneuploid genomes with nonrandom numerical changes in uneven ploidy ranges define distinct subsets of hematologic malignancies and solid tumors. The idea put forward herein suggests that they emerge from interactions between diploid mitotic and G0/G1 cells, which can in a single step produce all combinations of mono-, di-, tri-, tetra- and pentasomic paternal/maternal homologue configurations that define such genomes. A nanotube-mediated influx of interphase cell cytoplasm into mitotic cells would thus be responsible for the critical nondisjunction and segregation errors by physically impeding the proper formation of the cell division machinery, whereas only a complete cell fusion can simultaneously generate pentasomies, uniparental trisomies as well as biclonal hypo- and hyperdiploid cell populations. The term “somatic sex” was devised to accentuate the similarities between germ cell and somatic cell fusions. A somatic cell fusion, in particular, recapitulates many processes that are also instrumental in the formation of an abnormal zygote that involves a diploid oocyte and a haploid sperm, which then may further develop into a digynic triploid embryo. Despite their somehow deceptive differences and consequences, the resemblance of these two routes may go far beyond of what has hitherto been appreciated. Based on the arguments put forward herein, I propose that embryonic malignancies of mesenchymal origin with these particular types of aneuploidies can thus be viewed as the kind of flawed somatic equivalent of a digynic triploid embryo.

## Introduction

The term “aneuploidy” refers to any type of numerical deviation from a normal haploid or diploid set of chromosomes in germ or somatic cells that result from the simultaneous or successive gain or loss of single or multiple normal or abnormal chromosomes, respectively. Any such changes disrupt the intricate functional genomic balance of the affected cell and requires the corresponding adaption and reorganization of its transcriptional and metabolic performance (Pihan and Doxsey, 2003; Kops et al., 2005; Weaver and Cleveland, 2007; Gisselsson, 2011a; Sheltzer and Amon, 2011; Gordon et al., 2012; Vitre and Cleveland, 2012; Davoli et al., 2013; Danielsen et al., 2016; Rutledge and Cimini, 2016; Schukken and Foijer, 2018; Chunduri and Storchová, 2019; Simonetti et al., 2019). The ability to cope with such disturbances depends not only on the type of the particular involved chromosomes or chromosomal regions but also on the tissue of origin and the developmental stage of the affected cells. Together these factors determine then whether at all and how well the resultant offspring can overcome the particular genomic modification and cope with it. The less developed an affected cell is, the more flexible it can respond to and compensate such disturbances by modifying and adapting its epigenome, proteome as well as functional and metabolic organization accordingly.

The emergence of numerical abnormalities in somatic cells plays a crucial role in the initiation and development of virtually all types of malignancies (Duesberg et al., 1998; Weaver and Cleveland, 2007; Gisselsson, 2011a; Sheltzer and Amon, 2011; Gordon et al., 2012; Ben-David et al., 2014). They apparently arise either as an unfortunate by-product of accidental, environmentally triggered or genetically determined spindle symmetry abnormalities and segregation errors (Pihan and Doxsey, 2003; Kops et al., 2005; Weaver and Cleveland, 2006; Cimini, 2008; Gisselsson, 2008, 2011b; Vitre and Cleveland, 2012; Ben-David et al., 2014; Danielsen et al., 2016; Simonetti et al., 2019). These changes commonly provoke illegitimate recombination, replication and repair system errors, which together cause a wide-spread destabilization of the entire genome and consequently produce more or less complex structural rearrangements and additional small- and large-scale copy number changes. The necessity of affected cells to continuously counteract such genomic imbalances as well as to react to varying external conditions eventually results in their clonal diversification. Taken together, the mechanisms that lead to the formation of such aneuploid genomes in malignant diseases are as diverse as the outcome and ensuing consequences of their action.

The gain or loss of single and, even more so, multiple chromosomes that may even occur simultaneously, alters the expression of hundreds of genes and consequently also the intricate balance of proteins and their interactions in cellular networks (Hertzberg et al., 2007; Li et al., 2013; Ben-David et al., 2014; Durrbaum and Storchova, 2016; Antonarakis, 2017; Wangsa et al., 2019; Yang et al., 2019). For the maintenance of intracellular homeostasis such gross dysregulations pose a significant challenge. Cancer cells, in particular, manage this challenge astonishingly well and counteract its otherwise detrimental consequences by adjusting their metabolic and increased energy needs, which at the same time also helps them to adopt to additional environmental stresses. How well this can be achieved, varies widely between different human cells, tissues and tumor types (Weaver and Cleveland, 2007; Sheltzer and Amon, 2011). Aneuploidy may thus either decrease or increase the proliferative capacity and survival fitness of cells often in a quite paradoxical manner (Weaver and Cleveland, 2007; Sheltzer and Amon, 2011; Valind et al., 2013).

Of specific interest are those biologically and clinically distinct subsets of hematologic malignancies and solid tumors whose genomes are characterized by extraordinary stable chromosome counts in uneven ploidy ranges, including hyperhaploid, hyperdiploid (more than 52 chromosomes) near-triploid, near-pentaploid and near hexaploid ones (Table 1). Apart from the well-known embryonic malignancies in children, i. e., specific forms of B-cell precursor acute lymphoblastic leukemia (BCP-ALL), neuroblastoma, Wilms’ tumor and rhabdomyosarcoma, they are also seen to a varying extent in many types of adult malignancies, including myeloma, diverse sarcomas as well as breast, uterus, kidney, colon and thyroid tumors. Supplementary Table 1 provides a list of such neoplasms together with their preferentially involved chromosomes. These patterns indicate that the compatible chromosome combinations are strongly tissue of origin- but also differentiation-stage dependent, as for instance can be inferred from those encountered in hyperdiploid BCP-ALL (with extra chromosomes 4, 6, 10, 14, 17, 18, 21 and X) (Paulsson and Johansson, 2009) versus those in hyperdiploid plasma cell neoplasms (with extra chromosomes 3, 5, 7, 9, 11, 15, 19, 21 and X), respectively (Fonseca et al., 2004; Supplementary Table 1).

**TABLE 1:** Common features of malignancies with chromosome counts in the uneven ploidy range.

• Homogeneous and clonally stable karyotypes
• Prevalence of pure numerical chromosome abnormalities
• Recurrent, nonrandom and tissue of origin specific chromosome patterns
• Mono- or biclonal concurrence of hyperhaploid, hypodiploid and analogous hyperdiploid cell populations
• Simultaneous presence of mono-, di-, tri-, tetra-, and/or pentasomic chromosomes
• Tetrasomic chromosomes always result from duplication of both homologues (“2+2” pattern)
• Common occurrence of acquired uniparental di- and trisomies
• Occasional chromothripsis of single chromosomes

Although the initiating cause of such gross genomic changes remains a matter of ongoing speculations, the shared features of the ensuing karyotypes provide, despite their chromosomal heterogeneity, at least some important clues about the underlying principle that govern their formation (Table 1). Since aneuploid BCP-ALL forms are by far the best documented and explored entities of this kind of malignancies, I will use them in the following to outline these illuminating karyotype peculiarities in more detail (Paulsson and Johansson, 2009; Safavi and Paulsson, 2017; Carroll et al., 2019).

## Hyperdiploid, Hyperhaploid, and Hypodiploid Forms of BCP All as Prototypic Examples

Pure numerical chromosome changes are seen in more than a third of all childhood ALL cases with a B-cell precursor immunophenotype. Based on the respective modal chromosome number, such cases are subdivided into potentially bimodal hyperhaploid, low hypodiploid and hyperdiploid forms with somewhat arbitrary chromosome ranges between 24 to 31, 32 to 39 and 52 to 67 chromosomes, respectively, a categorization that is further vindicated by the biological and molecular features as well as clinical behavior of these particular sub-entities (Harrison et al., 2004; Heerema et al., 2007; Paulsson and Johansson, 2009; Holmfeldt et al., 2013; Muhlbacher et al., 2014; Safavi and Paulsson, 2017; Carroll et al., 2019).

The preeminent and connecting karyotypic feature of these three aneuploid categories is an overrepresentation of chromosome 21 either in form of a bi-parental disomy in hyperhaploid and hypodiploid cases or in form of a bi-parental tetrasomy in hyperdiploid ones. Additional numerical changes that define hyperdiploid cases are trisomies, most commonly those of chromosomes X, 4, 6 10, 14, 17 and 18, as well as tetrasomies, primarily of chromosomes X and 14 (Mertens et al., 1996; Heerema et al., 2007; Paulsson and Johansson, 2009). Trisomies usually result from the duplication of either one of the parental chromosomes in an apparently random fashion (“2+1” pattern), whereas tetrasomies always derive from the duplication of both parental homologues (“2+2” pattern). Extrapolating this duplication mechanism, one would thus expect six chromosomes 21 (“3+3” pattern) in hyperdiploid leukemias of patients with a constitutional trisomy 21. However, to the best of my knowledge, such a hexasomy 21 has not yet been documented (Forestier et al., 2008; Maloney et al., 2010). Instead, one still encounters only tetrasomies and occasionally, as may sometimes also be the case in constitutional normal cases, pentasomies of chromosome 21 in these instances. Hyperdiploid leukemias are underrepresented and neither hyperhaploid nor hypodiploid forms are known in individuals with a Down syndrome, which indicates that a preexistent trisomy 21 impedes the development of such leukemias, although it has no discernable effect on the ensuing hyperdiploid chromosome configuration after its manifestation (Forestier et al., 2008; Maloney et al., 2010). Moreover, the notion that pentasomies and occasionally hexasomies build a general upper limit of tolerable copy numbers is further supported by observations in near-triploid neuroblastomas (Kaneko and Knudson, 2000; Tomioka et al., 2003; Teixeira and Heim, 2005; Gisselsson et al., 2007) and embryonal rhabdomyosarcomas (Bridge et al., 2000; Teixeira and Heim, 2005), which usually contain only up to five and only exceptionally six chromosomes 17 and five copies of chromosomes 8, 11 and 13, respectively.

When viewed from a statistical point of view, individual chromosomes seem to appear in a predictable non-random fashion that depends on the ones already present as well as the overall modal number (Mertens et al., 1996; Heerema et al., 2007). Apart from the omnipresent tetrasomy 21, which is always the first change, chromosomes X, 14, 6, 18, 4, 17 and 10 are then acquired in an decreasing order of likelihood (Heerema et al., 2007; Paulsson and Johansson, 2009). These findings underline the notion that this hierarchical order reflects the required compatibility and interdependence of the vital interactions between the gained chromosomes.

Other intriguing albeit much rarer are neoplasms with hyperhaploid or hypodiploid clones that coexist with their hyperdiploid counterparts with analogous chromosome sets in an seemingly duplicated manner (Mandahl et al., 2012). Apart from BCP-ALL (Holmfeldt et al., 2013; Safavi et al., 2013; Carroll et al., 2019), such clone combinations are commonly seen in oncocytic forms of thyroid, parathyroid and adrenocortical carcinomas as well as occasionally also in chondrosarcomas, malignant fibrous histiocytomas and peritoneal mesotheliomas (Supplementary Table 1).

In case of ALL, such a biclonality has been reported in up to 64% of hyperhaploid and 44% of low-hypodiploid cases (Holmfeldt et al., 2013; Safavi et al., 2013; Carroll et al., 2019). The evident relationship of the respective genomes has led to the understandable and hitherto unchallenged view that in these instances a haploidization step must precede the subsequent formation of the respective hyperdiploid clone (Charrin et al., 2004; Paulsson and Johansson, 2009; Gisselsson, 2011a, b; Chen et al., 2013; Holmfeldt et al., 2013; Safavi et al., 2013; Baughn et al., 2015; Carroll et al., 2019; Lundin-Strom et al., 2020). Thus, chromosomes that are monosomic in the hyperhaploid/hypodiploid clones reappear as uniparental disomies in the hyperdiploid ones, whereas those which were originally biparental disomic retain their heterozygosity when they become tetrasomic. Such a copy neutral loss of homozygosity (CN-LOH; uniparental disomy) of entire chromosomes is also commonly encountered in “pure” hyperdiploid cases, whereas the triplication of a particular chromosome in form of an uniparental trisomy is much rarer (Lundin et al., 2016). Of note, chromosome 21 is always biparental disomic in the hyperhaploid/hypodiploid clones.

Irrespective of their closely related karyotypic features, hyperhaploid, hypodiploid and “pure” hyperdiploid leukemias acquire not only shared but also subset-specific distinct somatic mutations in genes that encode components of distinct signaling pathways (Supplementary Table 2). In “pure” hyperdiploid and hyperhaploid cases, for instance, they target mainly RTK/RAS pathway genes, such as *KRAS*, *NRAS*, *FLT3* and *PTPN11*, as well as histone modifiers that comprise apart from *CREBBP* also *WHSC1*, *SUV420H1*, *SETD2* and *EZH2* (Inthal et al., 2012; Malinowska-Ozdowy et al., 2015; Paulsson et al., 2015; de Smith et al., 2016). More typical for hyperhaploid forms alone, are those affecting *NF1*, *CDKN2A/B*, the 6p22 histone gene cluster, *IKZF3* and *PAG1* (Holmfeldt et al., 2013; Safavi et al., 2013). The hallmark of more than 90% of hypodiploid cases, on the other hand, are loss-of-function mutations in *TP53* which already preexist in approximately half of them in the germ line (Holmfeldt et al., 2013; Safavi et al., 2013).

Physiological B cell development follows discrete steps (van Lochem et al., 2004; Hystad et al., 2007; Lee et al., 2012; Bendall et al., 2014). It commences already during early fetal life and requires the ordered successive rearrangement of immunoglobulin genes (van Zelm et al., 2005; Jung et al., 2006). This process concurs with a massive expansion of immature precursor cells and a subsequent tight selection process that is only survived by those few cells that succeed to functionally rearrange their immunoglobulin genes (Jung et al., 2006). Such cell-specific rearrangements confirm the single cell origin of BCP leukemias and serve as valuable clonal markers for monitoring treatment efficacy.

The immunoglobulin heavy chain gene locus (*IGH*) is the first one that rearranges. It is located on chromosome 14, which incidentally is also the most frequent trisomic or even tetrasomic one in hyperdiploid BCP ALL (Panzer-Grumayer et al., 2002; Heerema et al., 2007; Csinady et al., 2009; Paulsson and Johansson, 2009). Because only one rearrangement can take place per *IGH* allele per cell, the maximum number of individual *IGH* rearrangements in such clones depends on the copy numbers of *IGH* alleles (Szczepanski et al., 2001; Panzer-Grumayer et al., 2002; Jung et al., 2006; Csinady et al., 2009). A clone with a disomy 14 can therefore only have a maximum of two unique rearrangements, whereas a clone with trisomy 14 could harbor either a maximum of three unique or one unique and two related rearrangements (Szczepanski et al., 2001). Thorough analyses of such rearrangement patterns indicate that the extra chromosome 14 is usually already present before the initiation of *IGH* recombination (Panzer-Grumayer et al., 2002; Csinady et al., 2009). Moreover, as can be inferred from the immunophenotype of such leukemias, the existence of extra copies of chromosome 14 freezes them in a more immature immunogenotypic stage than all other genetic BCP ALL subgroups (Csinady et al., 2009). Taken together, these observations provide clear evidence that the maldistribution of chromosomes is indeed the leukemia-initiating event.

## Formation of Uneven Ploidy Patterns

The core idea of the now already 30 years old and still endorsed model is that nonrandom uneven ploidy patterns are the outcome of hitherto unidentified genetic or epigenetic perturbations in one or several components of the cell division machinery, which eventually cause the maldistribution of chromosomes most likely in a single abnormal cell division (Paulsson et al., 2005). The mis-segregation mechanism itself is supposed to involve tripolar mitoses, sister chromatid nondisjunction and/or incomplete or asymmetric cytokinesis, essential factors that are part of four distinct but not mutually exclusive routes that eventually lead to a final stable karyotype (Paulsson et al., 2003; Gisselsson, 2008, 2011a, b; Paulsson and Johansson, 2009).

These routes comprise

(i) sequential sister chromatid nondisjunction events in consecutive cell divisions,
(ii) an initial loss of a near-haploid set of chromosomes with a subsequent duplication of the remaining ones,
(iii) an initial tetraploidization step followed by the loss of a near-haploid chromosome set and
(iv) a simultaneous gain of all the respective chromosomes in a single abnormal cell division.

Based on all the hitherto collected evidence, there is now generally agreement that route (i) is the most unlikely, whereas route (iv) seems to be the most probable one. To assess the likelihood of route (i), Gisselsson et al. applied an elaborate *in silico* modeling, which mimicked the parallel evolution of 500 tumor stem line karyotypes over 2.000 generations, however, without respecting any potential cell- or tissue-related interdependence of the various gained chromosomes (Gisselsson, 2011b). This approach provided apparently some vague evidence that the main contributors to the formation and maintenance of aneuploidy are an increased mitotic error rate and an elevated tolerance toward newly integrated whole chromosomes, but on the whole was not able to convincingly demonstrate that this is a feasible way to achieve stable aneuploidies (Gisselsson, 2011b).

The argument for the notion that all extra chromosomes are indeed gained simultaneously during an asymmetric cell division derives from the analyses of the allelic ratios of tetra- and disomic chromosomes (Paulsson et al., 2005). Based on the respective distribution, Paulsson et al. considered that “in all practice high hyperdiploidy arises either via a tetraploid pathway (approximately 30% of cases) or by a simultaneous gain (approximately 70% of cases)” (Paulsson and Johansson, 2009). Although multipolar mitosis, whose most common form is tripolar, could in principle foster such a simultaneous maldistribution of multiple chromosomes, it nevertheless seems unlikely that such a constellation would be able to allocate the chromatids of di- or tetraploid cells into three more or less equal, appropriately functioning portions rather than distribute them in a more random chaotic fashion (Stewenius et al., 2005; Gisselsson, 2008, 2011a, b; Valind et al., 2013).

Both routes (iii) and (iv), on the other hand, still require multiple convoluted and hypothetical steps to achieve the final form of aneuploidy. For instances, based on a computer-modeled random loss of chromosomes, Giselsson considered it highly unlikely that any such non-random homologue distribution patterns could evolve from a tetraploid state (Gisselsson, 2011a, b). So far, the strongest support that at least either one of these two tracks could indeed be taken derives from the co-existence of hyperhaploid and hyperdiploid clones with analogous distribution patterns of corresponding chromosomes.

The particular set of potentially defective genes that are of primary interest in the context of the nondisjunction model are especially those, whose products regulate the spindle assembly checkpoint and the sister chromatid cohesion/separation process. Germ line mutations cause, amongst others, the cancer-prone syndrome of premature chromatid separation with mosaic variegated aneuploidy (Hanks et al., 2006). The characteristic hallmarks of these syndromes are aneuploidies that occur in many tissues and involve chromosomes in a more or less random fashion (Hanks et al., 2006). Affected individuals have obvious dysmorphic features, experience a variety of clinical problems and have a high risk to develop malignancies, such as rhabdomyosarcoma, Wilms tumor and leukemia. Of note, although the chromosome pattern of an aneuploid embryonal rhabdomyosarcoma in one of these individuals was typical for this particular type of tumor, it differed from all those aneuploidies that emerged in non-malignant tissues (Hanks et al., 2006). With regard to the potential contribution of condensin complex and spindle assembly checkpoint impairments, Molina et al. found that inhibition of the Aurora B kinase and the spindle assembly checkpoint produced substantial chromosomal instability in healthy CD34-positive hematopoietic cells, the outcome of which were indeed aneuploid cells with chromosomes that displayed ALL-typical structural and condensation defects (Molina et al., 2020). In support of these findings, Moura-Castro et al. (2020) also observed similar severe cohesion defects in a large proportion of hyperdiploid cases, especially in those with an increased copy number heterogeneity. Nevertheless, even if one concedes that these observations are highly relevant, it still remains unclear what actually would trigger these disturbances in the first place.

## Partial or Complete Cell Fusions Cause Mononuclear- or Binuclear-Derived Aneuploidies

**FIGURE 1:**
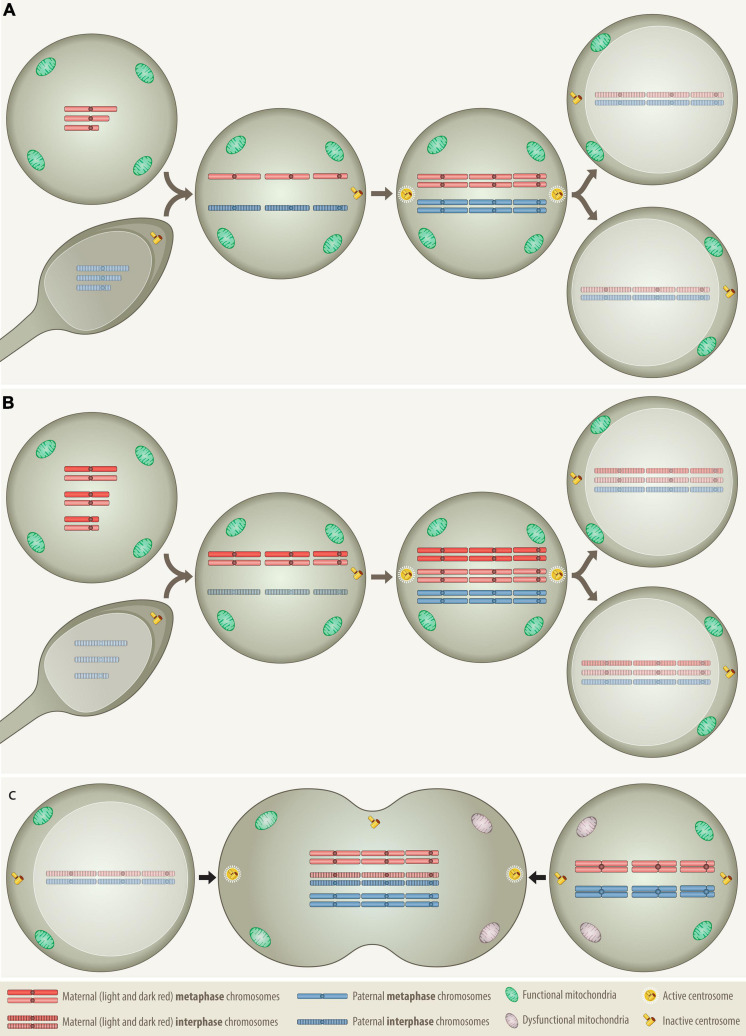
Comparison of the distribution patterns of chromosomes, centrosomes and mitochondria in three different types of cell fusion: **(A)** a haploid oocyte with a haploid sperm, **(B)** a diploid oocyte with a haploid sperm, and **(C)** a mitotic cell with a G0/G1 cell. Fertilization and somatic cell fusion are closely related processes that share many physical and functional properties. To enable the amalgamation of the nuclear material of the involved cells, at least one of the fusion partners needs to be in mitosis, because only a mitotic cell can supply the essential cytoplasmic components that promote the breakdown of nuclear membrane and condense the interphase nucleus into chromatid chromosomes. The combined set of chromosomes must then be accurately allocated to two daughter cells, which is best achieved with the help of a single pair of properly functioning centrosomes. Only daughter cells that receive at least the equivalent of a complete haploid set of chromosomes together with an adequate number of functional mitochondria can survive. **(A)** In case of normal fertilization, the haploid oocyte provides the power-supplying mitochondria as well as the essential hospital mitotic environment with all the essential components that are necessary to condensate the interphase nucleus of the intruding haploid sperm into individual chromatids, whereas the sperm contributes the prepared centrosome *Anlage*, which upon fusion is converted into properly functioning centrosomes (Clift and Schuh, 2013). Following duplication of both haploid sets of chromosomes the cell divides and generates the first pair of diploid daughter cells. **(B)** The formation of a digynic triploidy, which results from the fusion of a diploid oocyte with a haploid sperm, follows essentially the same track. Since digynic triploid zygotes also contain only one single pair of active centrosomes, they can engage in relatively normal mitosis so that all progenitor cells will inherit a stable triploid set of chromosomes. Owing to the consequences of meiotic recombination, homologous chromosomes in diploid oocytes may still contain distinguishable heterozygous regions (Hassold and Hunt, 2001). **(C)** The somatic type of cell fusion alluded to herein, fuses a mitotic with a G0/G1 interphase cell and resembles therefore in many ways the germline one that produces a digynic triploidy. As the meiotic environment condenses the sperm, the mitotic one will also condense the G0/G1 interphase nucleus into single-stranded chromatid chromosomes, a phenomenon that in the somatic setting is known as “premature chromosome condensation (PCC)” (Ravi et al., 2013). The outcome of this cell fusion is thus a transient hexaploid mitotic cell, with three maternal and three paternal sets of chromosomes (“3+3”), whereas its digynic germline counterpart comprises four maternal and two paternal sets (“2+2+2”). Although such a somatically fused cell is per definition tripolar, in terms of functionality it actually remains bipolar, since only the two centrosomes that derive from the mitotic cell are the operational active ones. As in the dyginic triploid zygote, this would in principle allow an appropriate reallocation of two complete triploid chromosome sets into daughter cells. However, such pure stable triploid cell populations can hardly be achieved, because fusions of somatic cells will always involve two already at least minimally differentiated cells with unequal epigenetic, metabolic and functional properties. Apart from the essential mitotic environment, the creation of viable progeny from such a fusion requires therefore the participation of two reasonably suited cell types that already contain and are able to contribute appropriate combinations of compatible and functionally interacting chromosomes to their offspring. Through this process, an appropriately fitted donor cell could likewise deliver fresh mitochondria to an energetically impaired mitotic cell and thereby increase the survival chances of the ensuing hybrid.

**FIGURE 2:**
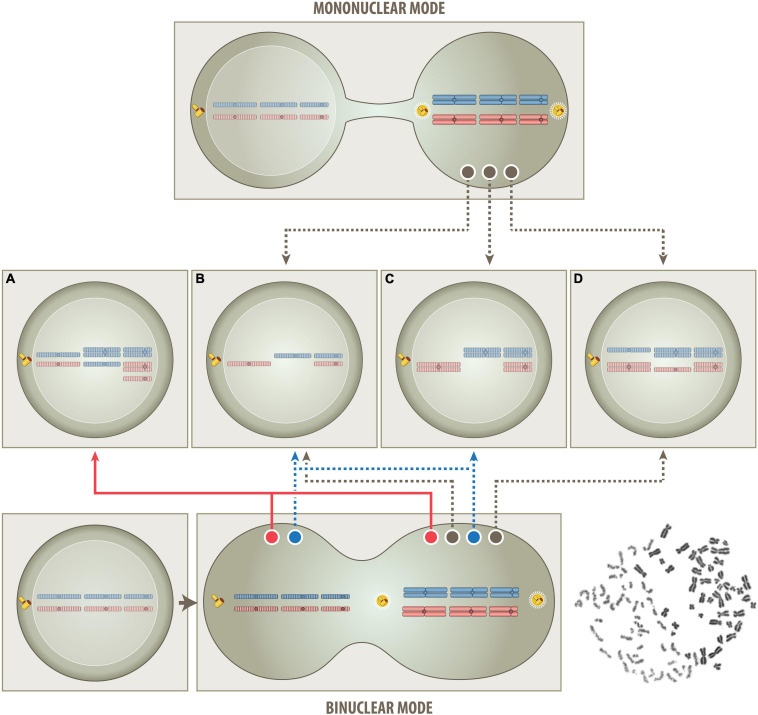
Schematic outline of the proposed one step routes that produce either a mononuclear- or binuclear-derived aneuploidy (MNDA & BNDA). In the mononuclear route (top part), all superfluous chromosomes derive solely from a single (tetraploid) mitotic cell. This model postulates that the untimely cytoplasmic influx from a non-dividing G0/G1 cell will disturb the phase separation and condensation process of various components of the cell division machinery (Ong and Torres, 2020) and thereby cause chromatid segregation and nondisjunction errors. The consistent heterozygous “2+2” pattern is not only a clear indication that tetrasomies always derive from both chromatids of a particular pair of metaphase chromosomes but, at the same time, also that they are the crucial and indispensable survival factor, since corresponding twin cells will lack both homologs and can therefore not survive. In case of BCP-ALL, for instance, the respective survival factor is a tetrasomy 21 (Paulsson and Johansson, 2009) and in case of oncocytic forms of thyroid carcinomas a tetrasomy 7 (Corver et al., 2012). Apart from the consistent occurrence of such “2+2” tetrasomy patterns, the mononuclear mode is consistent with and can therefore also easily explain all other patterns and combinations of chromosomes, as long as they only comprise between one and four copies and not more than two homologs of either one, i. e., monosomies, heterozygous di-, tri- and tetrasomies as well as homozygous (“uniparental”) disomies **(B–D)**. Of special note in this context is the unique form of hyperdiploidy that only consists of homozygous disomic and heterozygous tetrasomic chromosomes (**C** and Figure 3, left). According to the hitherto generally uphold view such patterns can only arise in consecutive steps that eventually duplicates an afore generated hyperhaploid **(B)** genome. Following this logic, such peculiar hyperdiploid patterns are therefore considered as a surrogate proof for the necessary existence of an originally hyperhaploid predecessor clone, irrespective of whether it can indeed be identified or not. The herein proposed tetraploid mitotic aneuploidization route, on the other hand, provides a plausible alternative explanation for how such hyperhaploid **(B)** or hyperdiploid **(C)** clones can derive from a single mitotic cell even in a single step. However, it also precludes that they can coexist in this particular setting since they cannot be produced simultaneously. Although this mononuclear route is very similar to the previously propagated tetraploid one, it differs in two important aspects, namely in the way the segregation of chromatids is affected and that only a single pair of centrosomes is operative and responsible for their subsequent allocation to daughter cells. During mitosis diploid cells become temporarily tetraploid, i.e., they contain 92 chromatid chromosomes and two functional centrosomes. Genuine tetraploid cells, on the other hand, may derive from diploid ones through endoreduplication, endomitosis or cell fusion, and therefore contain double the number of chromatids, chromosomes and centrosomes. They are a frequent transitory state on the route to aneuploidy and, once formed, can undergo either bipolar, tetrapolar, or tripolar divisions in the following mitotic cycles. Bipolar divisions will again produce two near-tetraploid cells; tetrapolar divisions four near-diploid cells and tripolar divisions in principle two near-triploid and one near-diploid daughter cells (Palitti and Rizzoni, 1972). Although it was proposed that a multipolar cell division combined with incomplete cytokinesis may be one way to generate a hyperdiploid karyotype, this remains overall only a rather remote possibility (Gisselsson et al., 2010; Gisselsson, 2011b). On the other hand, the development of digynic triploid embryonic tissues prove that, given the proper circumstances, two appropriately functioning centrosomes can even continuously distribute hexaploid intermediates into regular stable triploid daughter cells. Such triploid conceptuses can sometimes fully develop and occasionally even survive for several months after birth (Sherard et al., 1986; Joergensen et al., 2014; Toufaily et al., 2016; Akhlaghdoust et al., 2017). Since mitotic hyperdiploid ALL cells also contain only two centrosomes, the same mode of division enables likewise their continuous, stable and faithful propagation (Molina et al., 2020). In the binuclear route (bottom part), the condensation of the G0/G1 interphase nucleus into single-stranded chromatids will, analogous to what happens in the formation of a digynic triploid zygote (Figure 1B), primarily create two clusters of chromosomes, one with 46 bi-chromatid metaphase and the other one with 46 prematurely condensed interphase chromatid chromosomes, as shown in an example that was obtained from a polyethylene glycol-mediated fusion of a PHA-stimulated mitotic peripheral blood with a G0/G1 chronic lymphocytic leukemia cell (bottom right). The two chromosome clusters may remain isolated or exchange and/or mix some of their chromosomes before they separate again into independent cells. All aneuploidy patterns that can be produced via the mononuclear route **(B–D)** can in a similar fashion of course also be achieved via the binuclear route. However, there are particular chromosome constellations, such as uniparental homozygous trisomies and different types of pentasomies (**A** and Figure 3, right), whose extra chromosomes can only stem from a second cell, if one maintains that these patterns are indeed generated simultaneously (red track) in a single event rather than through subsequent ongoing missegregation processes. Moreover, this BNDA cell fusion model can also provide an immediate explanation for how hyperhaploid/hypodiploid **(B)** and their equivalent hyperdiploid **(C)** clones can be produced simultaneously (blue tracks). Although the matching chromosome patterns in the corresponding clones mimic a duplication event, these clones will actually derive from two different cells and may therefore also have distinguishable properties. In contrast to the previous multistep model that interprets the relationship of such corresponding clones somehow as the progeny of a sole individual cell, this particular single step model views them as same generation (fraternal) twins of two genuine parent cells. Further supporting this interpretation is also the fact that the number of chromosomes of hyperhaploid/hypodiploid clones together with their equivalent hyperdiploid counterparts never exceeds 138, a sum that makes up the predicted hexaploid set of chromatids in this particular type of somatic cell fusion.

**FIGURE 3:**
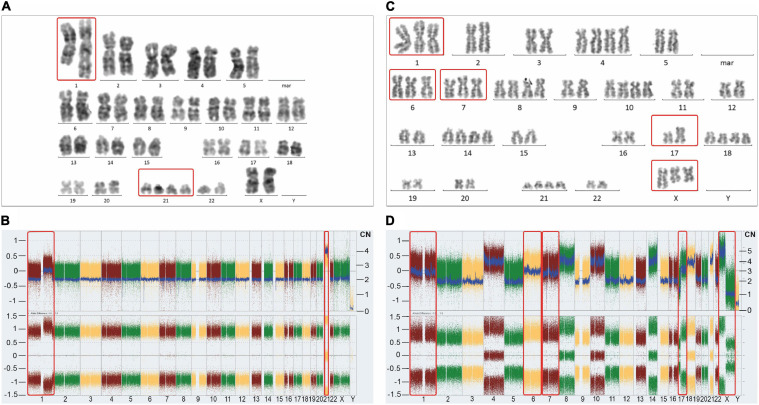
Representative karyotypes **(A,C)** and SNP/CGH array-derived whole genome views **(B,D)** of a postulated mononuclear-derived monoclonal **(A,B)** and a postulated binuclear-derived pure hyperdiploid ALL case **(C,D)**. Affymetrix Cytoscan HD SNP arrays were processed according to the manufacturer’s recommendation and analyzed with the ChAS Software (Affymetrix, Santa Clara, CA, United States). The weighted log2 ratio, shown in the top (y-axis, left scale) indicates the respective copy numbers (y-axis, right scale) and the allele difference plot in the bottom shows the SNP distribution patterns (y-axis; homozygous AA = 1, heterozygous AB = 0, homozygous BB = −1). The x-axis indicates the chromosome numbers. Of note, corresponding mono-and biclonal hyperhaploid and hyperdiploid cell populations produce indistinguishable array patterns, so that cytogenetic, DNA-index and/or FISH analyses are required to keep them apart and to verify their presence. The unique example on the left stems from a female patient and concords with the schematic outline in Figure 2C. Apart from the obligatory heterozygous tetrasomy 21 (red frame), which is the mandatory trademark of such aneuploidies, it only contains homozygous disomies. Would it be not for this extraordinary array pattern, this case with 48 chromosomes would neither be recognized nor considered as belonging to the aneuploid entities referred to herein. As validated with chromosome and FISH analyses, the 1q duplication is only present in approximately 50% of the leukemic cells, which together with its homozygous nature confirms its secondary nature. The instructive hyperdiploid example from a male patient with 62 chromosomes and a somatic *TP53* mutation on the right concords with the schematic outline in Figure 2A (kindly provided by Mayur Parihar, TATA Medical Center, Kolkata, India). Apart from exclusively homozygous disomies and heterozygous tetrasomies this case also contains homozygous trisomies of chromosomes 1, 6 and 7 (red frames). Provided one sticks to the one step mechanism of aneuploidization, the additional chromosomes cannot derive from the mitotic cell alone anymore. Moreover, since males have only one X, the presence of two isochromosomes Xp in addition to a normal one, can only mean that these two copies represent the co-segregated chromatids of an isochromosome Xp that already preexisted in the mitotic cell. The normal X can then only be the prematurely condensed one from the interphase cell. The isochromosome 17q, on the other hand, might have already preexisted before the fusion or evolved only thereafter. The Y chromosome was lost in this clone.

The model proposed herein is not only able to reconcile virtually all hitherto accumulated and partly contradicting findings, but it is also able to explain how any such aneuploidies can in principle be straightforwardly generated in a single step. Rather than evoking any particular type of *cell-intrinsic* causative genetic or epigenetic defects, it suggests that any such nonrandom aneuploidies result from an interaction of mitotic and a G0/G1 cells, either in form of a partial or complete cell fusion (Figures 1, 2). Although the extent of this cellular interaction may be a gradual continuum, its particular strength and length will eventually determine, which of two types of aneuploidies can be generated, namely one, in which all chromosomes derive exclusively from the mitotic cell alone (“mononuclear-derived aneuploidy; MNDA”), or one that also incorporates chromosomes from the nucleus of the respective G0/G1 fusion partner (“binuclear-derived aneuploidy; BNDA”) (Figure 2). Representative examples of such MNDA- and BNDA-derived karyotypes are shown in Figure 3.

Just based on the chromosome patterns alone, one can deduce that the most common hyperdiploid forms probably derive from a single, unequally dividing diploid mitotic cell (Figures 2B–D). In case of a partial cell fusion, the untimely and inappropriate influx and admixture of cytoplasmic material from an interphase cell into a mitotic one would thus interfere *physically* with phase separation and condensation processes that spatially and temporally regulate the assembly, formation and localization of crucial structures that normally guarantee a successful cell division (Ong and Torres, 2020). Such an interference would most likely affect the proper alignment of the chromosomes along the metaphase plate and/or impede the spindle apparatus and thereby obstruct the appropriate allocation of sister chromatids to the daughter cells (Morrison and Kimble, 2006; Schroeder, 2007; Lee and Vasioukhin, 2008; Silkworth and Cimini, 2012; Maiato and Logarinho, 2014; Farina et al., 2016; Akera et al., 2017; Niculescu, 2020; Ong and Torres, 2020).

**FIGURE 4:**
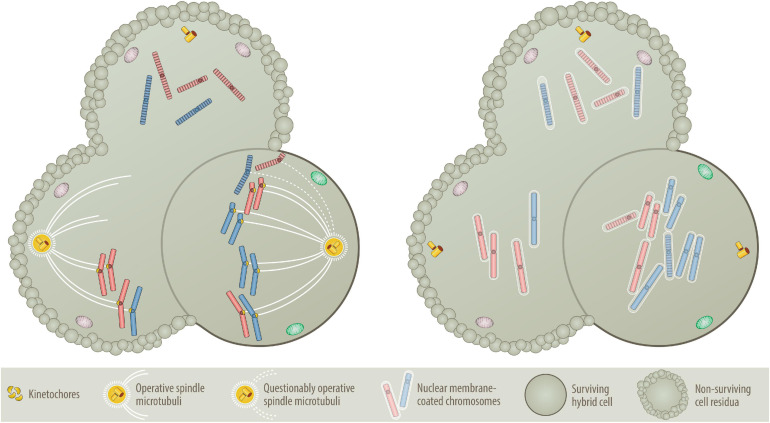
Nondisjunction **(Left)** versus “micronuclei” fusion **(Right)** versions of aneuploidy formation. One of the central questions of the BNDA model is whether at all and how prematurely condensed G0/G1 chromosomes can be incorporated into the mitotic chromosome cluster. As illustrated on the left, prematurely condensed chromosomes have no kinetochores, which are deemed necessary to facilitate their attachment to the microtubular spindle network (Tanaka et al., 2005; Cimini, 2008; Thompson and Compton, 2011; Silkworth and Cimini, 2012). As essential components of the mitotic spindle apparatus these multiprotein structures assemble only at the centromeres of sister chromatids in mitosis, where they control, supervise and coordinate sister chromatid segregation (Tanaka et al., 2005; Cimini, 2008; Silkworth et al., 2009; Thompson and Compton, 2011; Silkworth and Cimini, 2012). One circumstantial evidence that can be put forward as an explanation is that at least in the accommodating cytoplasmic milieu of an oocyte, prematurely condensed G0/G1 chromatids of somatic cells can replace sperm-derived ones and attain their segregation competence even without preexisting kinetochores (Saadeldin et al., 2016). Although the vast majority of such semi-somatic zygotes are aneuploid, they nevertheless can even go through early steps of embryonic development (Saadeldin et al., 2016). A further supportive argument resides on the commonplace method that is used to clone animals, in which interphase nuclei of various differentiated donor cells are transferred into empty, enucleated oocytes (Wells, 2005; Wilmut et al., 2015). Importantly, both procedures work only with G0/G1 cells and best with quiescent nuclei of G0 cells (Wells, 2005; Wilmut et al., 2015; Saadeldin et al., 2016). Another kinetochore- and spindle-unrelated mechanism (illustrated on the right) that in a pure somatic setting offers perhaps a much more plausible explanation, relates to the dissolution and reformation process of interphase nuclei (Burke and Ellenberg, 2002; Stewart et al., 2007; Guttinger et al., 2009; Hetzer, 2010; Lu et al., 2011; Schooley et al., 2012). When interphase nuclei split up into individual chromosomes, they produce abundant nuclear membrane fragments that are temporarily stored in the endoplasmatic reticulum before they are reused for reassembling the newly formed segregated chromosomes sets into new nuclei again (Guttinger et al., 2009; Lu et al., 2011; Schooley et al., 2012). To achieve this, membrane fragments first coat the individual chromosomes, which only then agglomerate and coalesce into distinct nuclei again (Schooley et al., 2012). Such single chromosome micronuclei form the basis for the microcell-mediated transfer of exogenous chromosome material into host cells (Meaburn et al., 2005; Fenech et al., 2011), which even enabled the successful integration of entire intact human chromosomes 8, 13, 18 and 21 into human isogenic embryonic stem cells (Kazuki et al., 2014; Hiramatsu et al., 2019) as well as chromosomes 3, 7 and 13 into the karyotypic stable, mismatch repair deficient colorectal cancer cell line DLD-1 (Upender et al., 2004; Nicholson et al., 2015; Rutledge and Cimini, 2016; Wangsa et al., 2019). A missegregated micronucleus can also undergo massive shattering and restructuring before these pieces rejoin again and form a new single chromatid, which can then become part of the nucleus again. Although this process, *chromothripsis*, is a frequent phenomenon in cancer genomes, it is rare in aneuploid leukemias (Chen et al., 2015). Presuming that this encapsulating mechanism will not discriminate between mitotic and prematurely condensed chromosomes, I consider this the most likely way of how the latter can get mixed up with mitotic ones, even without the necessity of evoking a microtubular-machinery for this purpose. Likewise, this mechanism seems also a reasonable way to explain how the remaining prematurely condensed chromosomes can rejoin again and form at the same time the genomic basis of a second independent aneuploid neoplastic founder cell provided they constitute at least a full haploid set, as illustrated in Figure 2B. The evolving aneuploid hybrid cell in the example shown here contains heterozygous tetrasomic, homozygous trisomic and pentasomic chromosomes, whereas the two other potential fusion cell descendants lack an adequate combination of chromosomes and can therefore not survive.

The relative timing of the influx and its spatial effect would then affect the distribution of the chromatids either in an already more or less deterministic, functionally fitting nonrandom or in a more indiscriminate random way, so that only those cells that obtain the essential compatible chromatid combinations will survive (Figure 4). In addition to extra chromosomes, the donor cell could also equip the recipient mitotic cells with cytoplasmic components that may help to promote the survival of the ensuing hybrid cell. Cases with a higher chromosome number, especially those with specific homologue distribution patterns and/or with two clones, where one is seemingly duplicated, defy such an explanation. Their chromosomal basis lies rather in the triploid than the diploid range. The only way to achieve such a triploid range in a single step is through a complete fusion of a mitotic with a G0/G1 cell, which under appropriate conditions would, as in digynic fertilization the replication of the newly formed trisomic chromosome set, produce a transient hexaploidy (Figure 1). A relative high proportion of such hexaploid sub-nuclei were seen, for instance, together with varying proportions of di-, tri-, tetra- and octaploid ones, in polyploid giant HeLa cells 4 days after irradiation (Salmina et al., 2019a).

Because of its likely futile outcome, the generation of aneuploid cells through fusion of mitotic or even G2 with G0/G1 cells has so far, if at all, only been envisaged as a far-fetched theoretical possibility. Still, the preconditions to achieve a single nucleus (synkaryon) in this situation is virtually the same as the ones that are also required for the unification of two separate interphase nuclei (homo- or heterokaryon) that would derive from the fusion of either two G0/G1 or two G2 cells from the same (homotypic) or different (heterotypic) tissues, respectively (Sottile et al., 2016). Interphase nuclei that start off in an asynchronous phase need to synchronize their cell cycle first to achieve a simultaneous coordinated mitotic stage during which they can merge their chromosomes, a process that is usually taken for granted but which has hardly ever been explored in any considerable detail (Sottile et al., 2016).

## Tunneling Nanotubes

Incomplete and complete cell fusions are two related processes with distinct purposes and consequences, whose involvement in the manifestation and progression of cancer is well established and widely accepted (Harris, 1988; Duelli and Lazebnik, 2007; Lu and Kang, 2009; Polak et al., 2015; Sisakhtnezhad and Khosravi, 2015; Bastida-Ruiz et al., 2016; Noubissi and Ogle, 2016; Platt et al., 2016; de Rooij et al., 2017; Vignais et al., 2017; Platt and Cascalho, 2019; Weiler and Dittmar, 2019). Given the high interest in these processes, it is thus surprising that they have hardly ever been considered to directly participate in the formation of a neoplastic founder cell. I am only aware of a single publication which showed that the homotypic fusion of non-transformed, cytogenetically stable rat epithelial intestine cells can initiate the transformation and the malignant outgrowth of a hybrid cell which, however, nevertheless concurred with considerable chromosome instability and DNA damage (Zhou et al., 2015).

TNTs, in particular, are thin filamentous F actin-based membrane channels, whose heterogeneous structures connect two or multiple cells, sometimes even in a network-like fashion and thereby form the fundamental basis of intercellular signaling and communication (Rustom et al., 2004; Marzo et al., 2012; Polak et al., 2015; Sisakhtnezhad and Khosravi, 2015; de Rooij et al., 2017; Nawaz and Fatima, 2017; Vignais et al., 2017; Pinto et al., 2020). They may either result from an actively extending cytoplasmic protrusion that eventually joins another cell or constitute cytoplasmic bridges that are left from an incomplete cytokinesis (Davis and Sowinski, 2008; Duarte et al., 2018; Lens and Medema, 2019). Depending on their length and diameter, such TNTs facilitate the electric coupling of cells as well as the unidirectional or bidirectional transport and exchange of ions, proteins and various organelles but also pathogens, such as viruses and bacteria (Rustom et al., 2004; Abounit and Zurzolo, 2012; Osteikoetxea-Molnar et al., 2016; Wang et al., 2018). TNTs, with albeit altered biochemical and biophysical properties, become particularly abundant in various disease states and stressful environments, such as virus infection, inflammation and cancer as well as *in vitro* serum-starved cells (Marzo et al., 2012; Sisakhtnezhad and Khosravi, 2015; Ariazi et al., 2017; Nawaz and Fatima, 2017).

A good example of what happens in an abnormal microenvironment is the nanotube-induced interactive signaling between ALL cells and mesenchymal stem cells (MSC), in which the latter are incited to release cytokines and chemokines. These factors then ensure the survival of the leukemic cells, not least because they also protect them from the noxious effects of cytotoxic drugs (Polak et al., 2015; de Rooij et al., 2017; Burt et al., 2019). Moreover, a nanotube-mediated transfer of mitochondria can even rescue damaged cells during a very early stage of apoptosis (Rustom et al., 2004; Wang and Gerdes, 2015; Dong et al., 2017; Griessinger et al., 2017; Herst et al., 2018; Burt et al., 2019). Conversely, prevention of nanotube formation and the mitochondrial transfer from activated mesenchymal stem cells with microtubule inhibitors, such as vincristine, enhances the therapy-induced apoptosis of adult ALL cells (Burt et al., 2019). Finally, nanotube connections may even instigate and set off proper cell fusion processes. In osteoclastogenesis, for instance, such F actin- and microtubule-containing TNTs, expedite the fusion of osteoclast precursors and thereby the formation of multinucleated cells (Takahashi et al., 2013).

One specific question that, to the best of my knowledge, has never been addressed previously, is how such TNT interactions would affect cells that either enter or are already in mitosis. In contrast to interphase cells, where the spatial location of the entry points of TNTs would probably not matter that much, their position could have detrimental consequences for mitotic cells. In addition to all the other above-mentioned metabolic readjustments that TNT-linked cells may have to deal with, the intrusion and influx of cytoplasmic material from a non-dividing cell could definitely affect the entire geometry of a mitotic cell with its otherwise orderly aligned chromosomes and its highly vulnerable intricate spindle scaffold (Ong and Torres, 2020). Depending on the site of the TNTs intrusion relative to the cell equator and depending on the magnitude of its exerted effect, such an interference could displace and move the metaphase plate with its accurately arranged chromatids and, consequently, also reposition the cell’s division plane and cytokinetic cleavage furrow in an analogous but much more subtle fashion as, for instance, was shown for asbestos fibers (Zhang et al., 2017; Ong and Torres, 2020). Together with the possible direct disruption of spindle tubules, such developments could ultimately lead to a maldistribution and irregular segregation of chromosomes into daughter cells, which are only able to survive and prosper in case they receive a proper fitting set of compatible chromosomes (Figure 4). Given that the mitotic spindle also defines the axis of a cell’s polarity, which codifies whether it will divide symmetrically or asymmetrically, the shift of a cell’s equatorial plane could therefore also alter its mode of division from symmetric to asymmetric or vice versa (Morrison and Kimble, 2006; Schroeder, 2007; Hawkins and Russell, 2008; Lee and Vasioukhin, 2008; Silkworth and Cimini, 2012; Maiato and Logarinho, 2014; Pham et al., 2014; Farina et al., 2016; Akera et al., 2017).

## Cell Fusion

Cell fusion is a universal process that plays a fundament role in the creation and propagation of life, in the physiological development, differentiation, repair and regeneration of tissues and organs but also in the formation and especially progression of neoplastic diseases (Ogle et al., 2005; Duelli and Lazebnik, 2007; Singec and Snyder, 2008; Lu and Kang, 2009; Dornen et al., 2020). Up to now, the vast majority of studies dealing with this phenomenon in cancer research primarily explored its role as a driving force in tumor progression (Lu and Kang, 2009; Mohr et al., 2015; Zhou et al., 2015; Noubissi and Ogle, 2016; Platt et al., 2016; Sottile et al., 2016; Searles et al., 2018; Platt and Cascalho, 2019; Weiler and Dittmar, 2019). The fusion of tumor cells with various types of tissue-related or bone marrow-derived ones, such as uncommitted stem cell-like progenitors or macrophages, produce genetically and phenotypically diverse hybrids (Sottile et al., 2016), whose extra dose of normal chromosomes is then supposed to instantly boost their evolutionary potential and equip them with novel, albeit unpredictable and therefore incalculable properties, such as enhanced drug resistance, migratory activity and homing ability (Skinner et al., 2012; Mohr et al., 2015; Sottile et al., 2016; Searles et al., 2018). Fusions of entire genomes and their subsequent reductive division (“sexual reproduction”) is deemed a much more efficient way to generate heterogeneous cell population than the continuous stepwise accumulation of oncogenic mutations in individual cells (“asexual reproduction”) (Zhang J. et al., 2014; Cofre and Abdelhay, 2017; Searles et al., 2018).

Despite the scrutiny and elegance of all these fusion experiments, they all remain surprisingly vague in explaining the necessary preconditions and processes that are required to merge, sort out, re-stabilize and functionally harmonize the genomes of the cell fusion product. In particular, it should be noted that the conversion of two interphase nuclei into a single one cannot simply be achieved by a direct fusion of nuclear membranes. Instead, the switch from any such homo- or heterokaryon into a synkaryon always requires the disassociation and reassembly of the nuclear envelopes of both cells, which means that both nuclei have to enter mitosis simultaneously, the only stage during which they can coalesce their chromosomes and redistribute suitable sets to viable mononuclear daughter cells (Sottile et al., 2016). Based on these premises, it is therefore understood that one requires two mitotic cells to start with. In case of normal diploid ones, their fusion will always create an octaploid intermediate and, if these 184 chromosomes properly segregate, two tetraploid daughter cells. It is thus easily overlooked that, as is the case in fertilization and premature chromosome condensation conditions, the cytoplasmic environment of a single meiotic/mitotic cell alone is sufficient to dissolve the nucleus of a fused interphase cell into individual chromatids, which may then replace the otherwise obligatory metaphase chromosomes (Figures 1, 4). Indirect support that indiscriminate cell fusion processes play indeed a role in such situations derives from observations in persistent polyclonal B lymphocytosis (Mossafa et al., 1999), where binucleated interphase cells frequently coexist with cells that undergo premature chromosome condensation and in chondrosarcomas, where they coincide with biclonal hyperhaploid and corresponding hyperdiploid cell populations (Olsson et al., 2011).

## … And Beyond: Re-Fusion, Entosis, Neosis, Meiomitosis, and Polyploidization

In addition to the above nongenetic routes to aneuploidy there are also several other fusion-related ones that deserve recognition, although they hardly can be envisioned as an initiating step in the creation of a neoplastic founder cell with the obligatory stable pure numerical aneuploidy patterns referred to herein.

One of these peculiar mechanisms is the *re-fusion* of sister cells that remain connected after an imperfect cytokinesis. Ongoing re-fusion events will then generate giant multinucleated cells, although the individual nuclei contained therein never merge (Rengstl et al., 2013). This particular process underlies the formation of Reed-Sternberg cells in Hodgkin’s lymphoma and, presumably also analogous types of multinucleated cells that are regularly encountered in other lymphoproliferative disorders, such as infectious mononucleosis, B-cell chronic lymphocytic leukemia, and T-cell lymphomas (Rengstl et al., 2013).

*Entosis*, on the other hand, is an intrusive non-apoptotic cell death mechanism, where the internalized cell is usually degraded by lysosomal enzymes (Overholtzer et al., 2007; Janssen and Medema, 2011; Krajcovic et al., 2011; Krajcovic and Overholtzer, 2012; Durgan et al., 2017; Garanina et al., 2017; Durgan and Florey, 2018; Mackay et al., 2018). It is a specific hallmark of normal as well as neoplastic epithelial cells that can take place when they detach, go through mitosis or are starved of glucose (Garanina et al., 2017). Consistent with the notion that this process is more a parasite-like invasion rather than a phagocytic engulfment, such internalized cells can not only survive for a certain period of time but also divide and even be expelled again. Although there is so far no evidence that the participating cells can fuse entirely nor that they can create a *bona fide* mononuclear hybrid, the interdependent physical interferences and cytokinetic obstructions that concur with the various dividing host and/or engulfed cell-in-cell configurations are nevertheless likely to produce progeny with unstable and aneuploid tumor-promoting genomes, especially when they take place in *TP53-*mutated cells (Janssen and Medema, 2011; Krajcovic et al., 2011; Durgan et al., 2017; Mackay et al., 2018).

Apart from cell fusion, polyploid cells can also be produced by distinct cell cycle oddities, such as *endocycling* and *endomitosis*, or a combination of both, which especially befall stressed cells (Sundaram et al., 2004; Erenpreisa et al., 2005; Walen, 2010; Krajcovic and Overholtzer, 2012; Orr-Weaver, 2015; Liu, 2020). Endocycling cells copy their genome by oscillating between a gap (G) and a DNA synthesis (S) phase without passing through a genuine mitotic (M) phase. In case of endomitosis, on the other hand, cells enter mitosis but cannot execute it properly, either because they assemble a spindle within a nucleus whose envelope does not break down, they are unable to correctly segregate sister chromatids or they cannot complete nuclear as well as cell division as needed (Sundaram et al., 2004; Erenpreisa et al., 2005; Walen, 2010; Krajcovic and Overholtzer, 2012; Orr-Weaver, 2015; Liu, 2020). In neoplastic cells these abnormal division processes often occur after a mitotic delay, when the respective cells divide without observing the necessary karyo- or cytokinetic corrective measures. In hyperdiploid ALL, for instance, mitotic slippage was observed in association with Aurora B kinase-impaired chromatid cohesion defects (Molina et al., 2020). In other circumstances, such a mitotic slippage usually leads to mitotic catastrophe and mitotic-delayed cell death. Rare cells that escape this fate turn into polyploid, phenotypically senescent cells. Depending on the respective trigger of the mitotic delay, they will upregulate not only their mitotic machinery but also reactivate a meiotic program and segregate their genetic material via multipolar and bipolar divisions into multiple large as well as small nuclei (Ianzini et al., 2009; Erenpreisa et al., 2011, 2015; Erenpreisa and Cragg, 2013; Lens and Medema, 2019). The latter can then detach and form again small mononuclear cells, which may still retain a certain life span and division capacity (Erenpreisa et al., 2005, 2011; Walen, 2005, 2007, 2010, 2014; Kalejs et al., 2006; Ianzini et al., 2009; Vitale et al., 2011; Zhang S. et al., 2014). One of the many labels which this series of events has received is *neosis* (Sundaram et al., 2004; Rajaraman et al., 2005). It is nowadays viewed as a kind of parasexual somatic reduction division, which transforms senescence cells into neoplastic ones and therefore plays and important role, especially in the formation, maintenance and progression of particular types of epithelial tumors. Analogous to meiotic recombination events, the reactivation of a meiotic program in mitotic somatic cells, *meiomitosis*, generates DNA double strand breaks that instigate repair activities, which in turn fabricate a plethora of structural rearrangements (Kalejs et al., 2006; Grichnik, 2008; Ianzini et al., 2009; Lindsey et al., 2013; Walen, 2014; Tsang et al., 2018; Salmina et al., 2019b).

## Cell-Fusion-Promoting (Micro)Environmental Prerequisites and Conditions

The preparedness of cells to unite via nanotubes or fusion depends on their specific operative organization and stage of differentiation as well as on their potential vital needs (Mohr et al., 2015). In case ensuing synkaryonic hybrids succeed indeed to reorganize their genomic structure and adapt their metabolic functions as required, their survival probability will henceforth rest on a hospitable microenvironment with feeder cells and supportive soluble products. Once established, the newly formed hybrid can then in turn begin to manipulate its niche according to its specific needs.

The most pronounced fusion-promoting venues are cell habitats with a high cell turnover, such as developing embryonic and regenerating tissues, wound healing, inflammation and infections, which at the same time contain substantial numbers of mitotic as well as apoptotic cells (Mohr et al., 2015; Noubissi and Ogle, 2016; Dornen et al., 2020). The fusogenic potential of such cellular ecosystems results from the hypoxic stress that is primarily exercised by the apoptotic cell fraction (Noubissi and Ogle, 2016). Given the disproportional high number of mitotic cells that are also present in such particular susceptible environments, one can thus expect that they will also get their fair chance to participate in fusion processes (Mohr et al., 2015; Bastida-Ruiz et al., 2016; Noubissi and Ogle, 2016). In a similar fashion, stagnant serum-starved *in vitro* cultures with an overcrowded cell number could in a comparable way increase the likelihood of spontaneous cell fusions and thereby be responsible for the common appearance of numeric aneuploidies in such systems (Li et al., 2000).

The vast majority of all childhood malignancies are initiated during early stages of embryonic tissue formation (Maris and Denny, 2002; Greaves et al., 2003; Greaves, 2005; Filbin and Monje, 2019). The specific cellular activities that take place during this developmental period make not only a perfect case for the unintended procreation of transforming gene fusions but also for aneuploidies (Greaves et al., 2003; Greaves, 2005). The probability of cell fusion activities may even be fueled further by additional cofounding factors, such as infections of the prospective mother with ubiquitous viruses or virus reactivation during pregnancy (Francis et al., 2017; Soegaard et al., 2018). Especially herpes viruses may thus incite and promote the development of cell fusion-related aneuploidies either directly through their specific fusogenic potential or indirectly through infection-associated inflammatory defense mechanisms (Parris, 2005b; Duelli and Lazebnik, 2007; Singec and Snyder, 2008; Gao and Zheng, 2011; Podbilewicz, 2014).

Because one cannot study these processes directly, one has to rely on traces and patterns that remain imprinted especially in the child’s immune system after birth and which at least can provide some indirect clues about what had happened prenatally. One of the most remarkable observations in this context was recently reported by Francis et al. (2017). Based on untargeted virome and bacterial analyses of pretreatment bone marrow specimens, the authors detected active cytomegalovirus transcription in leukemia blasts as well as intact virions in the serum of children with ALL, although the authors did unfortunately not provide any information about the genetic leukemia subtypes, which they had analyzed (Francis et al., 2017). Along this line, measurement of cytokines and inflammatory markers in the serum at diagnosis as well as retrospectively on neonatal dried blood confirmed that ALL children are already born with signs of dysregulated immune functions (Chang et al., 2011; Soegaard et al., 2018). Further indirect proxy measures for a genetically directed prenatal immune response derive from HLA segregation patterns in leukemic families and, in particular, the HLA types of the respective ALL patients (Taylor, 1994; Taylor et al., 2002, 2008). Based on such data, Taylor et al. concluded that BCP ALL is an indirect outcome of a transient auto-immune induced inflammatory molecular mimicry reaction that in turn may also explain why it appears to be associated with delayed infection (Taylor et al., 2008).

## Niches

The developmental opportunities of cells in which a transforming event takes place depends not only on their baseline epigenetic state that defines their differentiation stage and lineage commitment but also on the permissive milieu in which they are embedded (Filbin and Monje, 2019; Haigis et al., 2019). One of the most instructive yet underappreciated example of such a fusion- and cell survival-promoting microenvironment is the privileged compartment that contain the primordial germ cells (Hubner et al., 2003). Irrespective of whether they reside in a male or female body, they have the potential to develop into either sperm or oocytes when they receive the appropriate stimuli. Two of the respective malignancies that originate from primordial germ cells are mature teratomas and non-seminomatous testicular germ cell tumors. The fact that their genomes usually comprise only normal disomic chromosomes that, however, are overwhelmingly homozygous was taken as indication that these tumors derive from a parthenogenetic reactivation of oocytes or a fusion of haploid oocytes and sperms, respectively (Muretto et al., 2001; Lu et al., 2005). In one instance, the fusion is thus supposed to be facilitated by the initial coalescence of primary follicles into biovular ones, whereas in the other, some spermatocytes are supposed to acquire certain oocyte-specific features that would render them penetrable for other sperms, a notion that also suffices to explain the high risk of developing testicular germ cell tumors in phenotypic female but genetically male individuals (Muretto et al., 2001; Lu et al., 2005). The most thought-provoking issue of such postulated fusion processes is obviously that these phenomena completely blur and traverse the borders of germ and somatic cell domains (Grichnik, 2008).

Although it is virtually impossible to either directly examine the conditions or recreate the embryonic ecosystem in which the transforming events that eventually produce aneuploid leukemias occur, one might nevertheless at least obtain some ideas about the relevant contributing factors by looking at those, which remain even still important for the survival and proliferation of genuine leukemic cells. In the forefront of these stands the necessary continuous physical interaction of hyperdiploid leukemic cells with bone marrow mesenchymal stroma cells, be it through direct adhesion molecule-mediated cell contacts or the bi-directional shuttling of various cytoplasmic components via nanotubes (Manabe et al., 1992; Polak et al., 2015; Pal et al., 2016; de Rooij et al., 2017). Together with the secretion of proinflammatory cytokines, these mesenchymal stroma cells thereby shield the leukemic cells from otherwise detrimental effects, such as oxidative stress and chemotherapeutic interventions, and protect them from apoptotic cell death (Pal et al., 2016).

## Cancersphere – the Realm of Somatic Fertilization, Embryogenesis, and Pregnancy

The particular chromosome patterns and biological features of some embryonal neoplasms with hyper- and in particular, near-triploid neoplasms led me already many years ago to suggest that they might derive from residues of a constitutional triploid mosaic (Haas, 1996a, b). This speculation was soon after refuted on basis of the microsatellite-ascertained distribution patterns of maternal and paternal homologs in trisomic chromosomes (Paulsson et al., 2003). However, as can be deduced from the patterns shown in Figure 1, my chromatid-based model confirms that this could formally still be a feasible but nevertheless rather unlikely and only very remote option (Figures 1, 2). Nevertheless, it is intriguing to note that at least the idea that a “fertilization-like” somatic cell fusion process might underly the formation of such aneuploidies has lingered around and surfaced again recently in more polished versions (Vinnitsky, 2014; Erenpreisa et al., 2015; Liu, 2018, 2020; Salmina et al., 2019a). One of the supportive arguments descends from *in silico* analyses of the X and Y sex chromosome configuration patterns of 2.928 near-triploid karyotypes from 15 male malignant and five benign tumor entities (Vainshelbaum et al., 2019). The authors found that XXY was by far the most predominating pattern in all these cases, which was taken as indication that these aneuploidies must be generated in a similar fashion as digynic triploidies (Vainshelbaum et al., 2019). To form such aneuploid patters from original somatic diploid cells, the authors conceived a rather convoluted, albeit again not entirely impossible route, in which a kind of “gametogenic” reprogramming and activation of a pseudo-meiotic mechanism takes place (Vinnitsky, 2014; Erenpreisa et al., 2015; Liu, 2018, 2020; Salmina et al., 2019a). This would necessitate first the separation of the two parental genomes into separate cells, followed by the duplication of the entire haploid set of chromosomes in the female one before it would fuse again with the haploid male cell (Vinnitsky, 2014; Erenpreisa et al., 2015; Liu, 2018, 2020; Salmina et al., 2019a). Although there are some indications that in certain tumor types the co-expression of specific genes of especially the meiosis I machinery affects the normal mitotic process (Lindsey et al., 2013; Tsang et al., 2018), I consider it unlikely that such an interference alone can indeed set such a complicated development into motion and bring it to a successful end.

Still, fertilization, which is without doubt the best known and most successful form of cell fusion, provides us with all the elements and ingredients that are also highly relevant in case of somatic cell fusion (Figure 1). To accentuate the similarities between germline and somatic fusion in general, the latter has already previously been termed “somatic sex” (Duelli and Lazebnik, 2007; Parris, 2008). Despite their somehow deceptive differences and consequences, a thorough comparison of germline and somatic cell fusion processes reveals that especially in embryonic malignancies of mesenchymal origin their resemblance may go far beyond of what has been hitherto appreciated.

Morphogenesis and morphostasis are core concepts that unify development, regeneration and cancer (Levin, 2012). Embryogenesis and tumorigenesis, in particular, share many remarkable dynamic and malleable features, not least the developmental route from a single founder cell to specific tissue types. Already a long time before the dawn of molecular genetics, such observations led to the perception that these two processes are closely intertwined. Supported by thorough examinations, elegant experiments and a vast amount of newly accumulated data, this concept experienced a notable revival within the recent years and gained again more attention and credibility. As explained in considerable detail in several excellent reviews of this topic, carcinogenesis should thus be viewed as a process of reproduction gone awry rather than one of uncontrolled growth (Old, 2007; Duesberg et al., 2011; Vinnitsky, 2014; Erenpreisa et al., 2015; Cofre and Abdelhay, 2017; Liu, 2018, 2020; Niculescu, 2020). It is thus supposed to somatically recapitulate a gametic program that starts with a deregulated stem cell and continues along blastomere-like stages of embryonic development (Simpson et al., 2005; Old, 2007; Vinnitsky, 2014; Erenpreisa et al., 2015; Liu, 2018, 2020; Niculescu, 2020), attributes that Old eventually interpreted in a provocative but very instructive manner as “somatic pregnancy” (Old, 2007). Along this line, atavistic interpretations of molecular and metabolic tumor-inherent peculiarities even advocate that the inappropriate re-expression of ancient gene sets in tumor tissues reiterates evolutionary trajectories that follow bacterial, fungal, and protozoan means of cell reproduction (Bussey et al., 2017; Trigos et al., 2017; Niculescu, 2020). It is thus not surprising that this asexual recapitulation of an organism’s genesis is also understood as a trajectory that aims to generate a new *bona fide* autonomous biological (parasitic) species (Duesberg and Rasnick, 2000; Parris, 2005a; Duesberg et al., 2011; Vincent, 2011, 2012; Knauss and Klein, 2012; Greaves, 2015).

Irrespective of the different labels under which all these comparable concepts appear, its interpretation always relies on the same common denominator, the polyploid giant cancer cell (PGCC; Walen, 2005, 2007; Vinnitsky, 2014; Erenpreisa et al., 2015; Liu, 2018, 2020). The fundamental polyploidization and depolyploidization escape and survival mechanisms of these cells together with their reactivated meiosis- as well as embryogenesis-specific genetic programs supply all essential ingredients that permit the formation of cyst-like tumor structures, which in turn encase reproductive stem as well as differentiating somatic cells with an even maintained capability to still manufacture all three germ layers. As pointed out, PGCCs derive from damaged or aged mature somatic cells and are predominantly components of epithelial tissues and their ensuing anaplastic counterparts. The ongoing reshuffling of their genomes creates clonally diverse, rather messy combinations of numerical and structural chromosome abnormalities. Embryonic neoplasms, on the other hand, which apparently lack such PGCCs, occur primarily in children and young adults (Maris and Denny, 2002; Filbin and Monje, 2019; Liu, 2020). They are well differentiated tumors whose architecture closely resembles the mesenchymal tissues from which they derive (Maris and Denny, 2002; Filbin and Monje, 2019). Moreover, their genome is remarkable stable and usually comprises only pure numerical chromosome abnormalities.

Near-triploid genomes, in particular, are seen in many distinct types of adenomas, sarcomas as well as carcinomas (Vainshelbaum et al., 2019). Taking the “somatic pregnancy” hypothesis of tumor formation into consideration, Vainshelbaum et al. therefore set out to explore, whether such genomes could indeed result from a somatic, fertilization-like cell fusion event (Vainshelbaum et al., 2019). They extracted all male near-triploid karyotypes from Mitelman’s cytogenetic database and found that their predominant sex chromosome configuration was XXY (Mitelman et al., 2021). In line with a digynic triploid embryo, this combination therefore indicates that the respective genomes comprise two maternal and one paternal haploid sets of chromosomes and that both must therefore be created in an analogous fashion. In a subsequent paper the authors presented a model and results of experiments that aimed to further substantiate that such near-triploid genomes are indeed produced in the postulated manner (Salmina et al., 2019a).

Although I share the authors’ opinion that near-triploid genomes primarily derive from a fertilization-like somatic cell fusion event, I nevertheless consider it highly unlikely that, at least in case of the specific tumor types alluded to herein, this is usually accomplished in the rather complicated and convoluted manner, which the authors propose. Moreover, for reasons given above, a route that requires a GPCC to start with seems not at all to be the ideal source to attain the stable aneuploidies one encounters in embryonic neoplasms of mesenchymal origin. The crucial problem of all current pure *chromosome*-based cell fusion models rests on the assumption that a triploid cell can only be achieved through fusion of a haploid and a diploid cell. My *chromatid*-based version, on the other hand, which relies on the fusion of a normal diploid G0/G1 cell and a normal tetraploid mitotic one, avoids this obstacle. As one can easily infer from the schematic outline in Figure 1, this particular type of fusion provides via an intermediate hexaploidy not only a convenient one step short cut for the formation of triploid genomes, but it suffices in the somatic setting the production of two (fraternal) twin descendants with either miscellaneous non-identical or even seemingly identical chromosome combinations simultaneously.

## Genomic Consequences and Developmental Prospects of Fused Cells

The destiny of fused cells predominantly depends on how well they are able to regroup and attune their chromosomes. Stabilizing such newly formed genomes and creating a viable and productive founder cell is neither in the germline nor in the somatic setting such a simple and straightforward process as the schematic depictions in Figures 1, 2 might imply. In both instances, the genomes of the fused and soon after separating cells have to go through harsh reorganization, adaptation and Darwinian selection steps before the final successful establishment of their progeny, which in the somatic situation is undeniably much more challenging than in the germline one. However, as analyses of *in vitro* fertilization-derived embryos revealed, such chromosomal reorganization processes are already even quite chaotic in the first post-zygotic stages of normal development (Ledbetter, 2009; Vanneste et al., 2009). It was shown that only ten percent of *in vitro* fertilized conceptuses were completely normal, whereas the remainder were heterogeneous mosaics that consisted of cells with miscellaneous types of chromosome abnormalities (Ledbetter, 2009; Vanneste et al., 2009). An intriguing and noteworthy further parallel that at least conceptually links embryonic and the proposed PGCC-type tumor development is the emergence of a so-called morula 3 to 4 days after fertilization, a compacted mass of 16 totipotent cells with indistinguishable borders that subsequently matures further into a cavitated, fluid-filled blastocyst (Liu, 2018, 2020). In that sense, the asynchronous fusion of somatic mitotic with G0/G1 cells would be a similar prerequisite for the initiation of aneuploid neoplasms as the fertilization of a diploid oocyte with a haploid sperm is for the generation of triploid conceptuses. Taking all these well-documented established facts and phenomena into consideration, the fusion of two normal somatic cells can thus be viewed as an analogous essential first step for the development of mesenchymal neoplasms with stable aneuploidies as is the GPCC for the development of epithelial carcinomas. Moreover, such a somatic cell fusion of two otherwise normal cells also provides the final missing link that very nicely feeds into and also closes the gap in Liu’s dualistic “giant cell cycle” and “life code” model of tumor development, which postulates that the origin and development of neoplastic tissues is the unfortunate and unsuccessful attempt of somatic cells to recapitulate the sexual route of reproduction (Erenpreisa et al., 2015; Liu, 2018, 2020; Salmina et al., 2019a).

## Somatic Mutations, Predisposing Germline Mutations, and Sequence Variants

The types of aneuploidies discussed herein usually concur with and conceal molecular alterations, which comprise somatic as well as predisposing germline mutations and sequence variants (Supplementary Table 2). Apart from their potential relevance for disease development and clinical decision making, they also help to delineate and better define discrete leukemia sub-entities, provide important insights into the relative timing and order of their appearance as well as the relationship between mono- and biclonal cell populations (Holmfeldt et al., 2013; Safavi et al., 2013, 2015; Malinowska-Ozdowy et al., 2015; Paulsson et al., 2015; de Smith et al., 2016).

The majority of classical hyperdiploid ALL as well as mono- and biclonal hyperhaploid entities acquire somatic mutations in receptor tyrosine kinase/Ras (RTK-Ras) and phosphoinositide 3-kinase (PI3K)-signaling pathway as well as in histone modifier genes (Holmfeldt et al., 2013; Malinowska-Ozdowy et al., 2015; Paulsson et al., 2015; Safavi et al., 2015; de Smith et al., 2016). Typical for the latter are also alterations in the *NF1*, histone cluster, *IKZF3* and *PAG1* genes that are found in roughly 44%, 19%, 13% and 10% of cases, respectively (Holmfeldt et al., 2013; Safavi et al., 2015). In contrast, over 90% of mono- or biclonal hypodiploid cases harbor *TP53* mutations, approximately 50% of which preexist already in the germ line (Holmfeldt et al., 2013; Muhlbacher et al., 2014; Safavi et al., 2015; Qian et al., 2018). Nearly half of all *TP53*-mutated cases acquire additional *IKZF2* and *RB1* mutations (Holmfeldt et al., 2013). The close biological relatedness of the classical hyperdiploid and hyperhaploid ALL forms is further underlined by their similar gene expression profile, whereas hypodiploid cases form a distinct separate entity (Gu et al., 2019). Collectively, these findings support the view that, irrespective of their mono- or biclonal appearance, MNDAs as well as all non-*TP53*-mutated BNDAs will form one common biologic entity, whereas *TP53*-mutated BNDA cases with their dissimilar genomic characteristics will form another independent distinct one (Holmfeldt et al., 2013; Muhlbacher et al., 2014; Safavi et al., 2015; Safavi and Paulsson, 2017; Qian et al., 2018).

In apparent support of the “mutation first” hypothesis, several of the somatically mutated genes, such as *ETV6, TP53* and those affecting the RTK/Ras pathway, occasionally preexist already in the germ line (Holmfeldt et al., 2013; Muhlbacher et al., 2014; Safavi et al., 2015; Safavi and Paulsson, 2017). Together with several already well-defined single nucleotid polymorphisms (SNPs), such as those associated with the *ARID5B* and *CEBBPE* genes, they constitute distinct genetic factors that apparently predispose specifically to the development of hyperdiploid leukemias in a rather unique manner (Wiemels et al., 2016; Studd et al., 2017). Whereas preexisting germ line mutations are more commonly encountered in childhood than in adult leukemias (Muhlbacher et al., 2014), it is not yet known whether this is also true for predisposing SNPs. Nevertheless, purely somatic mutations are always inter- and intragenic heterogenous secondary events that characterize abundant and widely fluctuating subclones (Malinowska-Ozdowy et al., 2015; Paulsson et al., 2015). Based on the presumption that the vast majority of biallelic mutations should arise from the duplication of a mutation that took place on one allele prior to trisomy formation, Paulsson et al. determined and calculated the number of homologs of trisomic chromosomes that carry such mutations (Paulsson et al., 2015). They found a compelling prevalence of single allele mutations in trisomic as well as in homozygous disomic chromosomes, which was taken as indication that mutations usually appear only quite some time after the aneuploidization event has taken place (Paulsson et al., 2015). Likewise, Studd et al. showed that the specific *ARID5B* risk allele on chromosome 10 is duplicated twice as often as the non-risk allele in in the leukemic blasts of cases with a trisomy 10 (Studd et al., 2017). Whether this also applies to other predisposition SNP-containing trisomies, remains to be shown. One such candidate is the *CEBPE* gene on chromosome 14, which is not only one of the earliest appearing and most common tri- and tetrasomic one but, incidentally, also harbors the disease-relevant *IGH* gene (Szczepanski et al., 2001; Panzer-Grumayer et al., 2002; Csinady et al., 2009; Wiemels et al., 2016). Other interesting functional variants are present in the *CDKN2A* and *CDKN2B*-containing region on chromosome 9, which is frequently altered in many different ALL subtypes (Morison et al., 2002; Mullighan et al., 2007; Safavi et al., 2013; Xu et al., 2015; Hungate et al., 2016; Lundin et al., 2016). In hyperdiploid ALL forms, these changes consist of an acquired homozygosity of the complete or at least the relevant parts of chromosome 9 (Morison et al., 2002; Safavi et al., 2013; Lundin et al., 2016), which commonly concurs with a heterozygous deletion of both *CDKN2A* and *CDKN2B* or a homozygous deletion of *CDKN2A* alone (Mullighan et al., 2007; Safavi et al., 2013). Noteworthy, such deletions seem to preferentially eliminate the maternal allele (Morison et al., 2002).

Given that some of these mutations can exist already before or arise only after the initiating aneuploidization event, begs for the questions whether and how germ line mutations contribute to the aneuploidization itself and how they eventually influence the subsequent choice of survival-compatible chromosome configurations or, conversely, why particular chromosome configurations permit only specific mutation routes. One explanation derives from the fact that the fate of cells and consequently also the functional aspects of tumor development is not only determined by the type of mutations and the tissue in which they take place but also by the order of their appearance (Haigis et al., 2019; Levine et al., 2019). Cases in point are *TP53* inactivating mutations, which cells with an otherwise normal genome tolerate apparently without any discernable effects (Bunz et al., 2002; Ho et al., 2010; Levine, 2020). Even fusion-generated tetraploid fibroblasts that undergo bipolar mitosis remain chromosomally stable over many generations as long as p53 is depleted only after their manifestation, whereas those which derive from already p53-inactivated diploid cells go through a phase of a crisis of chromosome destabilization before they finally transform (Fujiwara et al., 2005; Ho et al., 2010). Tetraploid cells that result from virus-mediated cell fusions, on the other hand, may overcome the initial cell cycle arrest and start proliferating, because especially oncogenic viruses produce proteins that perturb the apoptotic, p53 as well as the retinoblastoma signaling pathways (Duelli et al., 2005; Gao and Zheng, 2011). Nonetheless, the unique relationship between *TP53* mutations and hypodiploid ALL is still quite puzzling, because any other similar types of aneuploid neoplasms are extremely rare in Li-Fraumeni syndrome individuals (Qian et al., 2018).

The vast majority of childhood leukemias evolve from transforming events that take place already *in utero* (Greaves et al., 2003; Maia et al., 2003, 2004; Greaves, 2005; Morak et al., 2013; Hein et al., 2020). Screening neonatal blood for the presence of leukemia-specific fusion genes confirmed that they are generated much more frequently than the corresponding leukemia incidence would indicate (Mori et al., 2002; Lausten-Thomsen et al., 2011; Zuna et al., 2011; Schafer et al., 2018; Hein et al., 2020). Based on the ample evidence provided so far, there is reason to believe that potentially aneuploidy-generating cell fusions must occur at least as often as the illegitimate recombination events that produce the typical leukemia-initiating fusion genes, a notion that is not least corroborated by the fact that hyperdiploid leukemias are the most common form of childhood ALL. In this scenario, predisposing genetic factors would therefore not trigger the aneuploidization event itself in a direct manner but just alleviate the manifestation of hybrid cells by equipping them *a priori* with essential pathway signaling and metabolic assets, which founder cells in non-predisposed individuals are forced to acquire only in a later stage. In other words, predisposing factors and cell fusions are primarily independent and unrelated but synergetic elements that merely concur and cooperate in unfortunate individuals, which immediately explains also their apparent intimate relationship.

## Diagnostic and Clinical Implications

Aneuploid childhood BCP leukemias with less than 46 and more than 52 chromosomes are traditionally subdivided into three categories, whereby those hyperdiploid ones that supposedly derive from a duplication event are sometimes confusingly also perceived as being hyperhaploid or hypodiploid, irrespective of whether such particular clones are indeed identified (Holmfeldt et al., 2013; Safavi et al., 2013, 2015). In general, the size of hyperhaploid clones is already much smaller than of their accompanying hyperdiploid ones. Not only are hyperhaploid mammalian cells proliferative disadvantaged, because their more pronounced chromosomal segregation problems require them to spend longer in mitosis, but because the activation of their p53-dependent cytotoxic response renders them also more likely to die (Olbrich et al., 2017).

Hyperhaploid or hypodiploid forms fare apparently significantly worse than the “classic” hyperdiploid ones as well as most other genetic subtypes (Raimondi et al., 2003; Harrison et al., 2004; Nachman et al., 2007; Carroll et al., 2019; Pui et al., 2019). Irrespective of their individual chromosome composition and divergent mutation patterns, haploid or hypodiploid cases are nowadays grouped together in all treatment studies. In some of them, they are stratified as *a priori* high-risk, whereas in others they are stratified based on their respective MRD levels, since it was shown that their outcome can also be substantially improved by MRD-guided intensive chemotherapy alone (Mullighan et al., 2015). Although the combined assessment of MRD, CGH array and mutation patterns clearly improves the biological categorization of aneuploid ALLs, such a revised classification does not entirely coincide with the hitherto chromosome number- and DNA content-based one anymore. One example is the peculiar hyperdiploid entity that, as CGH analyses revealed, contains only homozygous disomies and heterozygous tetrasomies (“2+4” pattern; Figures 2, 3). According to the predominant current view, such cases represent “duplicated hyperhaploid” ones, a notion that is then often extended to include also cases with multiple homozygous disomies, such as the one shown in Figures 3C,D, despite the fact that they are only part of hyperdiploidies that also contain heterozygous disomies and trisomies. My model is instead clearly able to rebuff this interpretation by providing a more plausible explanation for the origin of both these aneuploid forms.

Carroll et al. (2019) showed recently that of 115 analyzed cases approximately a third of the hyperhaploid and a quarter of the hypodiploid ones were monoclonal, whereas 40% of both categories were biclonal, i.e., they coexist with their corresponding hyperdiploid counterpart. Reevaluating all previously published 110 cases, they found that former studies had only included biclonal or monoclonal hyperhaploid or hypodiploid cases but had completely left out monoclonal (“duplicated”) hyperdiploid ones (Raimondi et al., 2003; Harrison et al., 2004; Nachman et al., 2007). This perplexing omission indicates that a considerable proportion (at least 25%) of cases that are in the current clinical limelight had not at all been included in these analyses, although the results of these studies serve now as basis for their current high-risk stratification in some treatment studies. Having not been recognized as something extraordinary, they were thus inadvertently assigned to the “classic” hyperdiploid group. Since the inclusion criteria of analyzed cases were only vaguely defined, it seems likely that the same biased classification and selection occurred also in the recent “Ponte di Legno” study of 306 hypodiploid cases, even more so, because these data were collected from 16 different study groups, which in any case use inhomogeneous definition criteria and ascertainment methods (Pui et al., 2019). Thus, in treatment studies that use cytogenetic, FISH and/or CGH/array analyses for their diagnostic ascertainment, cases with specific array-derived “2+4” allelic distribution patterns might be classified as “high-risk duplicated hyperhaploids”, whereas in those, which only use DNA index measurements alone they would not even be recognized as something special. Rather than being able to resolve at least some of the diagnostic classification issues, the refined portrayal of aneuploid childhood leukemias has simply exposed some of these dilemmas and added more confusion.

In line with the results of mutation analyses, my model implies that all “classical” as well as monoclonal “2+4” hyperdiploid and hyperhaploid cases are most likely part of the mononuclear-derived group, whereas the bi-nuclear derived biclonal ones form a separate entity. Whether the mutation spectrum in these two groups is the same or follows different tracks remains to be evaluated. The third big group would then comprise all *TP53*-mutated hypo- and hyperdiploid cases, all of which would be binuclear-derived, irrespective of whether they are mono-or biclonal. In these instances, it will be interesting to find out whether preexistent germ line and somatically acquired mutations influence the formation of particular chromosome, allelic or clonal distribution patterns and, not least, disease development in distinguishable ways.

## Experimental Verification

Although I tested the plausibility of my model by reviewing chromosome and array patterns of over 200 aneuploid childhood ALLs, this is of course insufficient to convincingly prove its validity. As alluded to above, any bulk analysis of biclonal cell populations, be it by phenotyping, array analysis or mutation screening are subject to conceal unique properties of smaller cell populations contained therein, especially if one is not even aware that despite their relatedness, they nevertheless could represent unique and distinguishable cell populations. The most important and relevant difference between the nondisjunction and the cell fusion model is that in the former biclonal populations are homotypic, i.e., they always derive from the same cell type, whereas in the later they would be heterotypic, i.e., they stem from two different cell types with distinct lineage features and most likely discernable immunophenotypic, epigenetic as well gene expression characteristics. It can therefore be expected that a more meticulous comparative examination of such biclonal cell populations, either by separating the individual clones or single cell analyses, will provide an appropriate answer (Caron et al., 2020). Moreover, it would also be worthwhile to retrospectively collect, reevaluate and compare the outcome of meticulously characterized mono- and biclonal forms independently.

The obvious way to directly prove the validity of the model presented herein would of course be to generate such hyperdiploid cell populations via the proposed heaxaploid/triploid route *in vitro*. Although fusing cells is in principle a straightforward process, the main challenges rest on the right choice of fusion partners and in keeping the ensuing hybrids alive and propagate them. As can already be inferred from the fact that even *bona fide* aneuploid leukemic cells hardly survive *ex vivo* without close contact and interaction with mesenchymal bone marrow stromal cells, this will be a considerable challenge and an extremely difficult task to accomplish (Manabe et al., 1992; Pal et al., 2016).

## Concluding Remarks

Pure and stable nonrandom numerical aneuploidies are enigmatic genomic alterations that have puzzled and confused researcher and clinicians since they were first discovered. Adding to this bewilderment are now novel findings, which, in particular, derive from array and mutation analyses that are virtually impossible to align with the decades-old nondisjunction model that still serves to explain their origin. The comprehensive and cohesive concept presented herein provides a novel view of how such aneuploid neoplasms might emerge and develop. It incorporates many recently explored and experimentally already well-defined genetic and biological elements, which enable the successful reinterpretation of hitherto amassed observations and collected empirical evidence concerning their origin and their unique genomic make-up. Despite being well-founded, I am aware that the arguments put forward herein are to a large extent only circumstantial. However, in keeping with Sonnenschein’s and Soto’s assertion that “*Theories and their principles are not only useful to provide explanations of biological phenomena, but also help in framing both in vivo, in culture, ex vivo and in silico experiments*” (Sonnenschein and Soto, 2016), I hope that the broad biological context in which this model is embedded will foster interest in and stimulate further research of this peculiar group of aneuploid neoplasms.

## Data Availability Statement

The original contributions presented in the study are included in the article/Supplementary Material, further inquiries can be directed to the corresponding author/s.

## Author Contributions

The author confirms being the sole contributor of this work and has approved it for publication.

## Conflict of Interest

The author declares that the research was conducted in the absence of any commercial or financial relationships that could be construed as a potential conflict of interest.

## Publisher’s Note

All claims expressed in this article are solely those of the authors and do not necessarily represent those of their affiliated organizations, or those of the publisher, the editors and the reviewers. Any product that may be evaluated in this article, or claim that may be made by its manufacturer, is not guaranteed or endorsed by the publisher.

## Glossary

**Table:**

“Somatic sex”	Process that fuses two somatic cells
Meiosis	Cell division process that produces haploid oocytes and sperm cells from diploid cells
Zygote	Cell that derives from the fusion of an oocyte and a sperm
Digynic triploidy	Genomes that contain one paternal and two maternal haploid sets of chromosomes
Mono-, di-, tri-, tetra-, and pentasomic	One to five homologous chromosomes
Heterozygous/homozygous	Two different or two identical alleles of a gene
Copy-neutral loss of heterozygosity	Originally heterozygous (parts of) disomic chromosomes become homozygous
Uniparental disomy	Both homologous of a pair of chromosomes derive from the same parent
Sister chromatids	Two identical linked copies of the same chromosome formed after DNA replication
Karyotype	An individual’s collection of chromosomes
Haploid, diploid, triploid, tetraploid, pentaploid, hexaploid, and octaploid	Either one to six or eight sets of 23 human chromosomes
Hyperhaploid	Genome with 24–30 chromosomes
Hypodiploid	Genome with less than 46 chromosomes (typically 33–44 chromosomes)
Hyperdiploid	Genome with more than 46 chromosomes (typically 50–67)
Centrosome	Organelle that serves as microtubule organizing hub
Di-, tri-, and multipolar	Mitosis with either two (normal), three or multiple centrosome hubs
Synkaryon	A nucleus that derives from the fusion of two preexisting nuclei
Homokaryon	A cell that contains two or more nuclei that derive from the fusion of cells from the same (homotypic) tissue (e.g., muscle cells)
Heterokaryon	A cell that contains two or more nuclei that derive from the fusion of cells from different (heterotypic) tissues (e.g., tumor and normal cells)
Cytokinesis	The part of the cell division process during which the cytoplasm divides into two daughter cells
Kinetochore	Protein structure associated with duplicated chromatids in eukaryotic cells where the spindle fibers attach during cell division
Isogenic	Genetically uniform, derived from the same embryonic tissue
Chromothripsis	Mutational process that produces thousands of clustered chromosomal rearrangements in a single event in localized and confined genomic regions in one or a few chromosomes
