# Supplementary material for: Somatic Sex: On the Origin of Neoplasms With Chromosome Counts in Uneven Ploidy Ranges
Source: Front Cell Dev Biol. 2021 Aug 4;9:631946. doi: 10.3389/fcell.2021.631946 (PMC8373647; doi:10.3389/fcell.2021.631946)
Supplement: Supplementary file 1 [file Data_Sheet_1.PDF]

Supplemental Table 1: Characteristics of aneuploid malignancies with primarily pure numerical chromosome abnormalities

Malignancy	Hyperhaploid/ Hypodiploid	Hyper-diploid	Near triploid	Bi-clonal	Overrepresented Chromosomes [a]	Underrepresented chromosomes [a]	Uniparental disomies [a,b]	References
B cell precursor acute lymphoblastic leukemia (BCP ALL)	+	+	+	+	X, 4, 6, 10, 14, 17, 18, 21		3, 5, 9	(1-14)
BCP ALL with t(9;22)/*BCR-ABL1* [c]					2, 19			(15)
BCP ALL with KMT2A rearrangements					X, 4, 6, 13, 21			(15)
BCP ALL with a (secondary) t(1;19)/*TCF3-PBX1* [d]					X, 4, 6, 10, 14, 17, 18, 21			(15)
Chronic myeloid leukemia	+	+		+				(15-17)
Plasma cell neoplasms	+	+	+		X, 3, 5, 7, 9, 11, 15, 19, 21	13, 14, 22		(18-22)
Acute myeloid leukemia (AML) [e]		+	+		4, 6, 8, 9, 10, 11, 13, 19, 21, 22			(7, 8, 23-25)
AML-M7					7, 8, 14, 19, 22			(26)
Neuroblastoma [f]			+		2, 6, 7, 8, 9, 10, 11, 12, 13, 17, 18, 20, 22	X, 3, 9, 10, 11, 15, 19		(27-34)
Embryonal rhabdomyosarcoma					2, 5, 6, 7, 8, 11, 12, 13, 19, 20			(32, 35, 36)
Wilms tumor		+			6, 7, 8, 9, 12, 13, 17, 20			(37)
Non-seminomateous germ cell tumors [g]							1-24	(38, 39)
Papillary renal cell carcinoma		+			3, 7, 12, 16, 17, 20			(32)
Chromophobe renal cell carcinoma	+					X, Y, 1, 2, 6, 10, 13, 17, 21		(32)

Breast, fibroadenoma		+			5, 11, 18, 20			(40)
Breast, phyllodes tumors		+			1, 4, 5, 6, 7, 10, 11, 12, 13, 16, 17, 18, 19, 20, 21		10, 11, 12	(41-43)
Uterine leiomyoma [f]		+			8, 12, 20, X		1-24	(44)
Thyroid oncocytic (Hürthle cell) adenoma/carcinoma	+	+		+	3, 4, 5, 7, 12, 17, 20			(45-48)
Parathyroid oncoytoma				+	5, 7, 9, 19, 20			(49)
Adrenocortical carcinomas	+		+	+	4, 5, 12	7		(49, 50)
Malignant fibrous histiocytomas	+		+	+	5, 18, 20, 21			(51),
Peritoneal mesothelioma								(52)
Chondrosarcoma	+	+	+	+	X, 2, 3, 5, 12, 15, 18, 20	10, 11, 14, 22		(53)
Colon adenomas [h]	+		+					(54)

[a] Owing to the scarce numbers of cases and/or vague and/or incomplete data that are available in some of these entities, the information provided herein can only provide a provisional overview.

[b] excludes UPDs ones in the hyperdiploid clones of biclonal cases

[c] *BCR-ABL1* can be a primary or secondary change

[d] *TCF3-PBX1* is a secondary change

[e] rare cases pentaploid

[f] occasionally penta- and hexasomy 17

[g] complete homozygous

[h] DNA index only

1. Charrin C, X Thomas, M Ffrench, QH Le, J Andrieux, MJ Mozziconacci, et al. A report from the LALA-94 and LALA-SA groups on hypodiploidy with 30 to 39 chromosomes and near-triploidy: 2 possible expressions of a sole entity conferring poor prognosis in adult acute lymphoblastic leukemia (ALL). Blood (2004) 104:2444-2451. doi: 10.1182/blood-2003-04-1299
2. Harrison CJ, AV Moorman, ZJ Broadfield, KL Cheung, RL Harris, G Reza Jalali, et al. Three distinct subgroups of hypodiploidy in acute lymphoblastic leukaemia. Br J Haematol (2004) 125:552-559. doi: 10.1111/j.1365-2141.2004.04948.x
3. Heerema NA, SC Raimondi, JR Anderson, J Biegel, BM Camitta, LD Cooley, et al. Specific extra chromosomes occur in a modal number dependent pattern in pediatric acute lymphoblastic leukemia. Genes Chromosomes Cancer (2007) 46:684-693. doi: 10.1002/gcc.20451
4. Paulsson K and B Johansson. High hyperdiploid childhood acute lymphoblastic leukemia. Genes Chromosomes Cancer (2009) 48:637-660. doi: 10.1002/gcc.20671
5. Holmfeldt L, L Wei, E Diaz-Flores, M Walsh, J Zhang, L Ding, et al. The genomic landscape of hypodiploid acute lymphoblastic leukemia. Nat Genet (2013) 45:242-252. doi: 10.1038/ng.2532
6. Safavi S, E Forestier, I Golovleva, G Barbany, KH Nord, AV Moorman, et al. Loss of chromosomes is the primary event in near-haploid and low-hypodiploid acute lymphoblastic leukemia. Leukemia (2013) 27:248-250. doi: 10.1038/leu.2012.227
7. Chilton L, RK Hills, CJ Harrison, AK Burnett, D Grimwade and AV Moorman. Hyperdiploidy with 49-65 chromosomes represents a heterogeneous cytogenetic subgroup of acute myeloid leukemia with differential outcome. Leukemia (2014) 28:321-328. doi: 10.1038/leu.2013.198
8. Chilton L, G Buck, CJ Harrison, RP Ketterling, JM Rowe, MS Tallman, et al. High hyperdiploidy among adolescents and adults with acute lymphoblastic leukaemia (ALL): cytogenetic features, clinical characteristics and outcome. Leukemia (2014) 28:1511-1518. doi: 10.1038/leu.2013.379
9. Paulsson K. Chromosome gains drive childhood ALL. Oncotarget (2015) 6:19360-19361. doi: 10.18632/oncotarget.5141
10. Safavi S, L Olsson, A Biloglav, S Veerla, M Blendberg, J Tayebwa, et al. Genetic and epigenetic characterization of hypodiploid acute lymphoblastic leukemia. Oncotarget (2015) 6:42793-42802. doi: 10.18632/oncotarget.6000
11. Paulsson K, H Lilljebjorn, A Biloglav, L Olsson, M Rissler, A Castor, et al. The genomic landscape of high hyperdiploid childhood acute lymphoblastic leukemia. Nat Genet (2015) 47:672-676. doi: 10.1038/ng.3301
12. Lundin-Strom KB, K Strom, A Biloglav, G Barbany, M Behrendtz, A Castor, et al. Parental origin of monosomic chromosomes in near-haploid acute lymphoblastic leukemia. Blood Cancer J (2020) 10:51. doi: 10.1038/s41408-020-0317-2
13. Lundin KB, L Olsson, S Safavi, A Biloglav, K Paulsson and B Johansson. Patterns and frequencies of acquired and constitutional uniparental isodisomies in pediatric and adult B-cell precursor acute lymphoblastic leukemia. Genes Chromosomes Cancer (2016) 55:472-479. doi: 10.1002/gcc.22349
14. Safavi S and K Paulsson. Near-haploid and low-hypodiploid acute lymphoblastic leukemia: two distinct subtypes with consistently poor prognosis. Blood (2017) 129:420-423. doi: 10.1182/blood-2016-10-743765
15. Paulsson K, CJ Harrison, MK Andersen, L Chilton, A Nordgren, AV Moorman, et al. Distinct patterns of gained chromosomes in high hyperdiploid acute lymphoblastic leukemia with t(1;19)(q23;p13), t(9;22)(q34;q22) or MLL rearrangements. Leukemia (2013) 27:974-977. doi: 10.1038/leu.2012.263
16. Andersson BS, M Beran, S Pathak, A Goodacre, B Barlogie and KB McCredie. Ph-positive chronic myeloid leukemia with near-haploid conversion in vivo and establishment of a continuously growing cell line with similar cytogenetic pattern. Cancer Genet Cytogenet (1987) 24:335-343. doi: 10.1016/0165-4608(87)90116-6
17. Gancberg D, JL Dargent, A Verhest, A Kentos, W Feremans, C Lohrisch, et al. Near haploid blast phase in a chronic myeloid leukemia detected by fluorescence in situ hybridization using a BCR-ABL probe. Cancer Genet Cytogenet (2001) 128:172-174. doi: 10.1016/s0165-4608(01)00403-4

18. Fonseca R, B Barlogie, R Bataille, C Bastard, PL Bergsagel, M Chesi, et al. Genetics and cytogenetics of multiple myeloma: a workshop report. Cancer Res (2004) 64:1546-1558. doi: 10.1158/0008-5472.can-03-2876
19. Avet-Loiseau H, C Li, F Magrangeas, W Gouraud, C Charbonnel, JL Harousseau, et al. Prognostic significance of copy-number alterations in multiple myeloma. J Clin Oncol (2009) 27:4585-4590. doi: 10.1200/JCO.2008.20.6136
20. Chng WJ and R Fonseca. Centrosomes and myeloma; aneuploidy and proliferation. Environ Mol Mutagen (2009) 50:697-707. doi: 10.1002/em.20528
21. Van Wier S, E Braggio, A Baker, G Ahmann, J Levy, JD Carpten, et al. Hypodiploid multiple myeloma is characterized by more aggressive molecular markers than non-hyperdiploid multiple myeloma. Haematologica (2013) 98:1586-1592. doi: 10.3324/haematol.2012.081083
22. Kristoffersson U, H Olsson, D Kelly, M Akerman and F Mitelman. Near-haploidy in a case of plasmocytoma. Cancer Genet Cytogenet (1986) 19:239-243. doi: 10.1016/0165-4608(86)90052-x
23. Luquet I, JL Lai, C Barin, L Baranger, C Bilhou-Nabera, E Lippert, et al. Hyperdiploid karyotypes in acute myeloid leukemia define a novel entity: a study of 38 patients from the Groupe Francophone de Cytogenetique Hematologique (GFCH). Leukemia (2008) 22:132-137. doi: 10.1038/sj.leu.2404974
24. Sandahl JD, J Abrahamsson and J Heldrup. Hyperdiploidy in childhood AML associated with low age and AML-M7. A NOPHO-AML study and literature review. Blood (2011) doi:
25. Lazarevic V, A Rosso, G Juliusson, P Antunovic, A Rangert-Derolf, S Lehmann, et al. Prognostic significance of high hyperdiploid and triploid/tetraploid adult acute myeloid leukemia. Am J Hematol (2015) 90:800-805. doi: 10.1002/ajh.24091
26. Tsujimoto H, S Kounami, Y Mitani, T Watanabe and K Takifuji. Neonatal Acute Megakaryoblastic Leukemia Presenting with Leukemia Cutis and Multiple Intracranial Lesions Successfully Treated with Unrelated Cord Blood Transplantation. Case Rep Hematol (2015) 2015:610581. doi: 10.1155/2015/610581
27. Kaneko Y, N Kanda, N Maseki, M Sakurai, Y Tsuchida, T Takeda, et al. Different karyotypic patterns in early and advanced stage neuroblastomas. Cancer Res (1987) 47:311-318. doi:
28. Kaneko Y and AG Knudson. Mechanism and relevance of ploidy in neuroblastoma. Genes Chromosomes Cancer (2000) 29:89-95. doi: 10.1002/1098-2264(2000)9999:9999<::aid-gcc1021>3.0.co;2-y
29. Tonini GP and M Romani. Genetic and epigenetic alterations in neuroblastoma. Cancer Lett (2003) 197:69-73. doi: 10.1016/s0304-3835(03)00081-8
30. Tomioka N, H Kobayashi, H Kageyama, M Ohira, Y Nakamura, F Sasaki, et al. Chromosomes that show partial loss or gain in near-diploid tumors coincide with chromosomes that show whole loss or gain in near-triploid tumors: evidence suggesting the involvement of the same genes in the tumorigenesis of high- and low-risk neuroblastomas. Genes Chromosomes Cancer (2003) 36:139-150. doi: 10.1002/gcc.10151
31. Betts DR, N Cohen, KE Leibundgut, T Kuhne, U Caflisch, J Greiner, et al. Characterization of karyotypic events and evolution in neuroblastoma. Pediatr Blood Cancer (2005) 44:147-157. doi: 10.1002/pbc.20179
32. Teixeira MR and S Heim. Multiple numerical chromosome aberrations in cancer: what are their causes and what are their consequences? Semin Cancer Biol (2005) 15:3-12. doi: 10.1016/j.semcancer.2004.09.006
33. Gisselsson D, G Lundberg, I Ora and M Hoglund. Distinct evolutionary mechanisms for genomic imbalances in high-risk and low-risk neuroblastomas. J Carcinog (2007) 6:15. doi: 10.1186/1477-3163-6-15
34. Lundberg G, Y Jin, D Sehic, I Ora, R Versteeg and D Gisselsson. Intratumour diversity of chromosome copy numbers in neuroblastoma mediated by on-going chromosome loss from a polyploid state. PLoS One (2013) 8:e59268. doi: 10.1371/journal.pone.0059268

35. Anderson J, A Gordon, K Pritchard-Jones and J Shipley. Genes, chromosomes, and rhabdomyosarcoma. Genes Chromosomes Cancer (1999) 26:275-285. doi:
36. Pandita A, M Zielenska, P Thorner, J Bayani, R Godbout, M Greenberg, et al. Application of comparative genomic hybridization, spectral karyotyping, and microarray analysis in the identification of subtype-specific patterns of genomic changes in rhabdomyosarcoma. Neoplasia (1999) 1:262-275. doi: 10.1038/sj.neo.7900036
37. Kullendorff CM, M Soller, T Wiebe and F Mertens. Cytogenetic findings and clinical course in a consecutive series of Wilms tumors. Cancer Genet Cytogenet (2003) 140:82-87. doi: 10.1016/s0165-4608(02)00635-0
38. Bussey KJ, HJ Lawce, SB Olson, DC Arthur, DK Kalousek, M Krailo, et al. Chromosome abnormalities of eighty-one pediatric germ cell tumors: sex-, age-, site-, and histopathology-related differences--a Children's Cancer Group study. Genes Chromosomes Cancer (1999) 25:134-146. doi:
39. Lu YJ, J Yang, E Noel, S Skoulakis, T Chaplin, M Raghavan, et al. Association between large-scale genomic homozygosity without chromosomal loss and nonseminomatous germ cell tumor development. Cancer Res (2005) 65:9137-9141. doi: 10.1158/0008-5472.CAN-05-1697
40. Rizou H, G Bardi, M Arnaourti, N Apostolikas, K Sfikas, A Charlaftis, et al. Metaphase and interphase cytogenetics in fibroadenomas of the breast. In Vivo (2004) 18:703-711. doi:
41. Dal Cin P, P Pauwels, P Moerman, H Qi and H Van Den Berghe. Hyperdiploidy in benign breast lesions. Cancer Genet Cytogenet (1998) 101:162-163. doi: 10.1016/s0165-4608(97)00259-8
42. Adeyinka A, F Mertens, I Idvall, L Bondeson and N Pandis. Multiple polysomies in breast carcinomas: preferential gain of chromosomes 1, 5, 6, 7, 12, 16, 17, 18, and 19. Cancer Genet Cytogenet (1999) 111:144-148. doi: 10.1016/s0165-4608(98)00233-7
43. Walther C, D Gisselsson, L Magnusson, J Nilsson, D Grabau, CM Kullendorff, et al. Biphasic, hyperdiploid breast tumors in children: a distinct entity? J Pediatr Hematol Oncol (2013) 35:64-68. doi: 10.1097/MPH.0b013e3182752877
44. Holzmann C, DN Markowski, D Koczan, BM Helmke and J Bullerdiek. Genome-wide acquired uniparental disomy as well as chromosomal gains and losses in an uterine epithelioid leiomyoma. Mol Cytogenet (2014) 7:19. doi: 10.1186/1755-8166-7-19
45. Corver WE and H Morreau. Unique landscape of widespread chromosomal losses in Hurthle cell carcinoma. Endocr Relat Cancer (2019) 26:L1-L3. doi: 10.1530/ERC-18-0481
46. Gopal RK, K Kubler, SE Calvo, P Polak, D Livitz, D Rosebrock, et al. Widespread Chromosomal Losses and Mitochondrial DNA Alterations as Genetic Drivers in Hurthle Cell Carcinoma. Cancer Cell (2018) 34:242-255 e245. doi: 10.1016/j.ccell.2018.06.013
47. Ganly I, V Makarov, S Deraje, Y Dong, E Reznik, V Seshan, et al. Integrated Genomic Analysis of Hurthle Cell Cancer Reveals Oncogenic Drivers, Recurrent Mitochondrial Mutations, and Unique Chromosomal Landscapes. Cancer Cell (2018) 34:256-270 e255. doi: 10.1016/j.ccell.2018.07.002
48. Dettori T, DV Frau, ML Lai, S Mariotti, A Uccheddu, GM Daniele, et al. Aneuploidy in oncocytic lesions of the thyroid gland: diffuse accumulation of mitochondria within the cell is associated with trisomy 7 and progressive numerical chromosomal alterations. Genes Chromosomes Cancer (2003) 38:22-31. doi: 10.1002/gcc.10238
49. Corver WE, T van Wezel, K Molenaar, M Schrumpf, B van den Akker, R van Eijk, et al. Near-haploidization significantly associates with oncocytic adrenocortical, thyroid, and parathyroid tumors but not with mitochondrial DNA mutations. Genes Chromosomes Cancer (2014) 53:833-844. doi: 10.1002/gcc.22194
50. Zheng S, AD Cherniack, N Dewal, RA Moffitt, L Danilova, BA Murray, et al. Comprehensive Pan-Genomic Characterization of Adrenocortical Carcinoma. Cancer Cell (2016) 29:723-736. doi: 10.1016/j.ccell.2016.04.002
51. Aspberg F, F Mertens, HC Bauer, J Lindholm, F Mitelman and N Mandahl. Near-haploidy in two malignant fibrous histiocytomas. Cancer Genet Cytogenet (1995) 79:119-122. doi: 10.1016/0165-4608(94)00124-t

52. Sukov WR, RP Ketterling, S Wei, K Monaghan, P Blunden, P Mazzara, et al. Nearly identical near-haploid karyotype in a peritoneal mesothelioma and a retroperitoneal malignant peripheral nerve sheath tumor. Cancer Genet Cytogenet (2010) 202:123-128. doi: 10.1016/j.cancergencyto.2010.07.120
53. Bovee JV, M van Royen, AF Bardoel, C Rosenberg, CJ Cornelisse, AM Cleton-Jansen, et al. Near-haploidy and subsequent polyploidization characterize the progression of peripheral chondrosarcoma. Am J Pathol (2000) 157:1587-1595. doi: 10.1016/S0002-9440(10)64796-7
54. Staarmann J, WF Kotb and I Petersen. DNA ploidy and morphology of colon tumors in the adenoma-carcinoma sequence. Folia Histochem Cytobiol (2015) 53:11-18. doi: 10.5603/FHC.a2015.0001
