# Supplementary material for: Somatic Sex: On the Origin of Neoplasms With Chromosome Counts in Uneven Ploidy Ranges
Source: Front Cell Dev Biol. 2021 Aug 4;9:631946. doi: 10.3389/fcell.2021.631946 (PMC8373647; doi:10.3389/fcell.2021.631946)
Supplement: Supplementary file 2 [file Data_Sheet_2.PDF]

Supplemental Table 2: Molecular genetic features of three distinct subtypes of aneuploid childhood B cell precursor ALL cases

Chromosome region	Candidate genes	SNP [a] / defect	(GV)/ (GL)/ (SL)[b]	Function	Hyper-diploid	Hyper-haploid[c]	Hypo-diploid[d]	References
1p13.2	*NRAS*	mutations	SL	RTK RAS[e]	+	+		(1-7)
2p22.1	*SOS1*	mutations	GL	RTK RAS	+			(1)
2q34	*IKZF2*	mutations	SL	lymphoid transcription factor			+	(3)
3p21.31	*SETD2*	mutations	SL	histone methyltransferase	+			(2-4)
4p16.3	*NSD2 (WHSC1)*	mutations	SL	histone methyltransferase	+		+	(2, 5)
5p14.2	*PRDM9*	rare allele	GV	histone methyltransferase, meiotic recombination	+			(8, 9)
5q31.1	*C5orf56*	rs886285	GV	lncRNA	+			(10)
6p21.31	*BAK1*	rs210143	GV	pro-apoptotic BCL2 antagonist	+			(10)
6p22	Histone cluster	deletion	SL	DNA replication and packaging		+		(3)
7p12.2.	*IKZF1*	deletions	SL	lymphoid transcription factor	+	+		(3, 6, 11, 12)
7q36.1	*EZH2*	mutations	SL	histone methyltransferase	+			(2-4)
8q21.13	*PAG1*	deletions	SL	Ras signaling inhibitor		+		(3)
9p21.3	*(TLE1)*	rs76925697	GV	transcription co-repressor	+			(10)
9p21.3	*CDKN2A & CDKN2B[f]*	rs3731217 rs77728904	GV	cyclin-dependent kinase inhibitors	+			(13-15)
9p21.3	*CDKN2A & 2B[f]*	deletions[g]	SL	cyclin-dependent kinase inhibitors	+	+	+	(10, 11, 13, 14)
10p12.2	*BMI1*	rs7088318	GV	chromatin remodeling, epigenetic repressor				(16, 17)
10q21.2	*ARID5B*	rs7090445	GV	histone demethylase, transcription coactivator	+			(18-21)
10p12.2	*PIP4K2A*	rs2296624	GV	PI-3-kinase regulator	+			(16, 17)
11q13.2	*KMT5B (SUV420H1)*	mutations	SL	transcription regulator, histone methyltransferase	+			(2, 3)
11q14.1	*GAB2*	mutation	GL	PI-3-kinase regulator	+			(22)
12p12	*KRAS*	mutations	SL	RTK RAS	+	+		(1-5, 7)

12p13.2	*ETV6*	multiple SNPs	GV	ETS transcription factor, transcription repressor	+	+	+	(23, 24)
12p13.2	*ETV6*	mutations deletions[f]	GL[h] & SL	ETS transcription factor, transcription repressor	+	+		(23-28)
12q24.1	*PTPN11*	mutations	GL[i] & SL	RTK RAS	+	+		(1) (2, 4, 5)
13q12.2	*FLT3*	mutations duplications	SL	RTK RAS	+	+	+	(1-5, 29, 30)
13q12.2	*PAN3*	deletions	SL[j]	RTK RAS	+	+	+	(31)
13q14.2	*RB1*	mutations deletions	SL	transcription factor, negative cell cycle regulator	+			(3, 6)
14q11.2	*CEBPE*	rs2239630	GV	transcription activator	+	+		(10, 18, 20, 21, 32)
16p13.3	*CREBBP*	mutations deletions	GL[k] & SL	transcription coactivator			+	(2, 4-6, 11)
17p13.1	*TP53*	mutations deletions	GL[l] & SL	transcription factor, cell cycle regulator		+		(3, 6, 33-35)
17q11.2	NF1	mutations deletions	GL & SL	RTK RAS	+			(2, 3)
17q21.1	*IKZF3*	haplotype	GV	lymphoid transcription factor		+	+	(32)
17q21.1	*IKZF3*	deletions	SL	lymphoid transcription factor	+			(3)
19p13.3	*DOT1L*	mutation	SL	histone methyltransferase	+			(4)
21q22.2	*ERG*	rs9976326	GV	ETS transcription factor, transcription regulator				(10)

[a] SNP:single nucelotid polymorphism [b] GV: germ line variant, GV: germline lesions, SL: somatic lesions [c] mono or biclonal hyperhaploid and/or corresponding hyperdiploid version [d] mono or biclonal hypoploid and/or corresponding hyperdiploid version [e] RTK RAS: component of the receptor tyrosine kinase signaling pathway [f] generic B & T ALL predisposing SNPs [g] very common in virtually all types of B & T-ALL [h] *ETV6*-linked leukemia predisposition and familial thrombocytopenia syndrome [i] Noonan syndrome-type rasopathy [j] *FLT3* upregulation via *PAN3* deletion-associated enhancer hijacking [k] Rubinstein-Taybi syndrome [l] Li-Fraumeni syndrome

1. Cave H, A Caye, M Strullu, N Aladjidi, C Vignal, A Ferster, et al. Acute lymphoblastic leukemia in the context of RASopathies. Eur J Med Genet (2016) 59:173-178. doi: 10.1016/j.ejmg.2016.01.003
2. Paulsson K, H Lilljebjorn, A Biloglav, L Olsson, M Rissler, A Castor, et al. The genomic landscape of high hyperdiploid childhood acute lymphoblastic leukemia. Nat Genet (2015) 47:672-676. doi: 10.1038/ng.3301
3. Holmfeldt L, L Wei, E Diaz-Flores, M Walsh, J Zhang, L Ding, et al. The genomic landscape of hypodiploid acute lymphoblastic leukemia. Nat Genet (2013) 45:242-252. doi: 10.1038/ng.2532
4. de Smith AJ, J Ojha, SS Francis, E Sanders, AA Endicott, HM Hansen, et al. Clonal and microclonal mutational heterogeneity in high hyperdiploid acute lymphoblastic leukemia. Oncotarget (2016) 7:72733-72745. doi: 10.18632/oncotarget.12238
5. Malinowska-Ozdowy K, C Frech, A Schonegger, C Eckert, G Cazzaniga, M Stanulla, et al. KRAS and CREBBP mutations: a relapse-linked malicious liaison in childhood high hyperdiploid acute lymphoblastic leukemia. Leukemia (2015) 29:1656-1667. doi: 10.1038/leu.2015.107
6. Groeneveld-Krentz S, MP Schroeder, M Reiter, MJ Pogodzinski, HJ Pimentel-Gutierrez, R Vagkopoulou, et al. Aneuploidy in children with relapsed B-cell precursor acute lymphoblastic leukaemia: clinical importance of detecting a hypodiploid origin of relapse. Br J Haematol (2019) 185:266-283. doi: 10.1111/bjh.15770
7. Wiemels JL, Y Zhang, J Chang, S Zheng, C Metayer, L Zhang, et al. RAS mutation is associated with hyperdiploidy and parental characteristics in pediatric acute lymphoblastic leukemia. Leukemia (2005) 19:415-419. doi: 10.1038/sj.leu.2403641
8. Hussin J, D Sinnett, F Casals, Y Idaghdour, V Bruat, V Saillour, et al. Rare allelic forms of PRDM9 associated with childhood leukemogenesis. Genome Res (2013) 23:419-430. doi: 10.1101/gr.144188.112
9. Woodward EL, ML Olsson, B Johansson and K Paulsson. Allelic variants of PRDM9 associated with high hyperdiploid childhood acute lymphoblastic leukaemia. Br J Haematol (2014) 166:947-949. doi: 10.1111/bjh.12914
10. Vijayakrishnan J, M Qian, JB Studd, W Yang, B Kinnersley, PJ Law, et al. Identification of four novel associations for B-cell acute lymphoblastic leukaemia risk. Nat Commun (2019) 10:5348. doi: 10.1038/s41467-019-13069-6
11. Inthal A, P Zeitlhofer, M Zeginigg, M Morak, R Grausenburger, E Fronkova, et al. CREBBP HAT domain mutations prevail in relapse cases of high hyperdiploid childhood acute lymphoblastic leukemia. Leukemia (2012) 26:1797-1803. doi: 10.1038/leu.2012.60
12. Stanulla M, E Dagdan, M Zaliova, A Moricke, C Palmi, G Cazzaniga, et al. IKZF1(plus) Defines a New Minimal Residual Disease-Dependent Very-Poor Prognostic Profile in Pediatric B-Cell Precursor Acute Lymphoblastic Leukemia. J Clin Oncol (2018) 36:1240-1249. doi: 10.1200/JCO.2017.74.3617
13. Xu H, H Zhang, W Yang, R Yadav, AC Morrison, M Qian, et al. Inherited coding variants at the CDKN2A locus influence susceptibility to acute lymphoblastic leukaemia in children. Nat Commun (2015) 6:7553. doi: 10.1038/ncomms8553
14. Hungate EA, SR Vora, ER Gamazon, T Moriyama, T Best, I Hulur, et al. A variant at 9p21.3 functionally implicates CDKN2B in paediatric B-cell precursor acute lymphoblastic leukaemia aetiology. Nat Commun (2016) 7:10635. doi: 10.1038/ncomms10635
15. Vijayakrishnan J, M Henrion, AV Moorman, B Fiege, R Kumar, MI da Silva Filho, et al. The 9p21.3 risk of childhood acute lymphoblastic leukaemia is explained by a rare high-impact variant in CDKN2A. Sci Rep (2015) 5:15065. doi: 10.1038/srep15065

16. Walsh KM, AJ de Smith, AP Chokkalingam, C Metayer, GV Dahl, LI Hsu, et al. Novel childhood ALL susceptibility locus BMI1-PIP4K2A is specifically associated with the hyperdiploid subtype. Blood (2013) 121:4808-4809. doi: 10.1182/blood-2013-04-495390
17. Xu H, W Yang, V Perez-Andreu, M Devidas, Y Fan, C Cheng, et al. Novel susceptibility variants at 10p12.31-12.2 for childhood acute lymphoblastic leukemia in ethnically diverse populations. J Natl Cancer Inst (2013) 105:733-742. doi: 10.1093/jnci/djt042
18. Papaemmanuil E, FJ Hosking, J Vijayakrishnan, A Price, B Olver, E Sheridan, et al. Loci on 7p12.2, 10q21.2 and 14q11.2 are associated with risk of childhood acute lymphoblastic leukemia. Nat Genet (2009) 41:1006-1010. doi: 10.1038/ng.430
19. Trevino LR, W Yang, D French, SP Hunger, WL Carroll, M Devidas, et al. Germline genomic variants associated with childhood acute lymphoblastic leukemia. Nat Genet (2009) 41:1001-1005. doi: 10.1038/ng.432
20. Prasad RB, FJ Hosking, J Vijayakrishnan, E Papaemmanuil, R Koehler, M Greaves, et al. Verification of the susceptibility loci on 7p12.2, 10q21.2, and 14q11.2 in precursor B-cell acute lymphoblastic leukemia of childhood. Blood (2010) 115:1765-1767. doi: 10.1182/blood-2009-09-241513
21. Studd JB, J Vijayakrishnan, M Yang, G Migliorini, K Paulsson and RS Houlston. Genetic and regulatory mechanism of susceptibility to high-hyperdiploid acute lymphoblastic leukaemia at 10p21.2. Nat Commun (2017) 8:14616. doi: 10.1038/ncomms14616
22. de Smith AJ, G Lavoie, KM Walsh, S Aujla, E Evans, HM Hansen, et al. Predisposing germline mutations in high hyperdiploid acute lymphoblastic leukemia in children. Genes Chromosomes Cancer (2019) 58:723-730. doi: 10.1002/gcc.22765
23. Moriyama T, ML Metzger, G Wu, R Nishii, M Qian, M Devidas, et al. Germline genetic variation in ETV6 and risk of childhood acute lymphoblastic leukaemia: a systematic genetic study. Lancet Oncol (2015) 16:1659-1666. doi: 10.1016/S1470-2045(15)00369-1
24. Nishii R, R Baskin-Doerfler, W Yang, N Oak, X Zhao, W Yang, et al. Molecular Basis of ETV6-Mediated Predisposition to Childhood Acute Lymphoblastic Leukemia. Blood (2020) doi: 10.1182/blood.2020006164
25. Topka S, J Vijai, MF Walsh, L Jacobs, A Maria, D Villano, et al. Germline ETV6 Mutations Confer Susceptibility to Acute Lymphoblastic Leukemia and Thrombocytopenia. PLoS Genet (2015) 11:e1005262. doi: 10.1371/journal.pgen.1005262
26. Noetzli L, RW Lo, AB Lee-Sherick, M Callaghan, P Noris, A Savoia, et al. Germline mutations in ETV6 are associated with thrombocytopenia, red cell macrocytosis and predisposition to lymphoblastic leukemia. Nat Genet (2015) 47:535-538. doi: 10.1038/ng.3253
27. Duployez N, W Abou Chahla, S Lejeune, A Marceau-Renaut, G Letizia, T Boyer, et al. Detection of a new heterozygous germline ETV6 mutation in a case with hyperdiploid acute lymphoblastic leukemia. Eur J Haematol (2018) 100:104-107. doi: 10.1111/ejh.12981
28. Karastaneva A, K Nebral, A Schlagenhauf, M Baschin, R Palankar, H Juch, et al. Novel phenotypes observed in patients with ETV6-linked leukaemia/familial thrombocytopenia syndrome and a biallelic ARID5B risk allele as leukaemogenic cofactor. J Med Genet (2020) 57:427-433. doi: 10.1136/jmedgenet-2019-106339
29. Taketani T, T Taki, K Sugita, Y Furuichi, E Ishii, R Hanada, et al. FLT3 mutations in the activation loop of tyrosine kinase domain are frequently found in infant ALL with MLL rearrangements and pediatric ALL with hyperdiploidy. Blood (2004) 103:1085-1088. doi: 10.1182/blood-2003-02-0418
30. Stam RW, ML den Boer, P Schneider, M Meier, HB Beverloo and R Pieters. D-HPLC analysis of the entire FLT3 gene in MLL rearranged and hyperdiploid acute lymphoblastic leukemia. Haematologica (2007) 92:1565-1568. doi: 10.3324/haematol.11220
31. Yang M, S Safavi, EL Woodward, N Duployez, L Olsson-Arvidsson, J Ungerback, et al. 13q12.2 deletions in acute lymphoblastic leukemia lead to upregulation of FLT3 through enhancer hijacking. Blood (2020) 136:946-956. doi: 10.1182/blood.2019004684

32. Wiemels JL, KM Walsh, AJ de Smith, C Metayer, S Gonseth, HM Hansen, et al. GWAS in childhood acute lymphoblastic leukemia reveals novel genetic associations at chromosomes 17q12 and 8q24.21. Nat Commun (2018) 9:286. doi: 10.1038/s41467-017-02596-9
33. Qian M, X Cao, M Devidas, W Yang, C Cheng, Y Dai, et al. TP53 Germline Variations Influence the Predisposition and Prognosis of B-Cell Acute Lymphoblastic Leukemia in Children. J Clin Oncol (2018) 36:591-599. doi: 10.1200/JCO.2017.75.5215
34. Safavi S, L Olsson, A Biloglav, S Veerla, M Blendberg, J Tayebwa, et al. Genetic and epigenetic characterization of hypodiploid acute lymphoblastic leukemia. Oncotarget (2015) 6:42793-42802. doi: 10.18632/oncotarget.6000
35. Muhlbacher V, M Zenger, S Schnittger, S Weissmann, F Kunze, A Kohlmann, et al. Acute lymphoblastic leukemia with low hypodiploid/near triploid karyotype is a specific clinical entity and exhibits a very high TP53 mutation frequency of 93%. Genes Chromosomes Cancer (2014) 53:524-536. doi: 10.1002/gcc.22163
